# Managing clinical uncertainty in older people towards the end of life: a systematic review of person-centred tools

**DOI:** 10.1186/s12904-021-00845-9

**Published:** 2021-10-22

**Authors:** Clare Ellis-Smith, India Tunnard, Marsha Dawkins, Wei Gao, Irene J. Higginson, Catherine J. Evans, on behalf of SPACE, Ellis-Smith, Yi, Gao, Norton, Marshall, Banerjee, Goodman, Higginson, Sube Banerjee, Marsha Dawkins, Clare Ellis-Smith, Catherine J. Evans, Claire Goodman, Irene J. Higginson, Matthew Maddocks, Christine Norton, David Seamark, India Tunnard, Colin Vincent, Gao Wei, Deokhee Yi

**Affiliations:** 1 King’s College London, Cicely Saunders Institute of Palliative Care, Policy and Rehabilitation, Bessemer Road, London, SE5 9PJ UK; 2 Guys & St Thomas’ NHS Foundation Trust, Westminster Bridge Road, London, SE1 7EH UK; 3 King’s College Hospital NHS Foundation Trust, London, UK; 4 Sussex Community NHS Foundation Trust, Brighton General Hospital, Elm Grove, Brighton, UK

**Keywords:** Review, Uncertainty, Aged, Palliative care, Process assessment (health care), Patient outcome assessment, Patient care planning, Advance care planning, Communication

## Abstract

**Background:**

Older people with multi-morbidities commonly experience an uncertain illness trajectory. Clinical uncertainty is challenging to manage, with risk of poor outcomes. Person-centred care is essential to align care and treatment with patient priorities and wishes. Use of evidence-based tools may support person-centred management of clinical uncertainty. We aimed to develop a logic model of person-centred evidence-based tools to manage clinical uncertainty in older people.

**Methods:**

A systematic mixed-methods review with a results-based convergent synthesis design: a process-based iterative logic model was used, starting with a conceptual framework of clinical uncertainty in older people towards the end of life. This underpinned the methods. Medline, PsycINFO, CINAHL and ASSIA were searched from 2000 to December 2019, using a combination of terms: “uncertainty” AND “palliative care” AND “assessment” OR “care planning”. Studies were included if they developed or evaluated a person-centred tool to manage clinical uncertainty in people aged ≥65 years approaching the end of life and quality appraised using QualSyst. Quantitative and qualitative data were narratively synthesised and thematically analysed respectively and integrated into the logic model.

**Results:**

Of the 17,095 articles identified, 44 were included, involving 63 tools. There was strong evidence that tools used in clinical care could improve identification of patient priorities and needs (*n* = 14 studies); that tools support partnership working between patients and practitioners (*n* = 8) and that tools support integrated care within and across teams and with patients and families (*n* = 14), improving patient outcomes such as quality of death and dying and satisfaction with care. Communication of clinical uncertainty to patients and families had the least evidence and is challenging to do well.

**Conclusion:**

The identified logic model moves current knowledge from conceptualising clinical uncertainty to applying evidence-based tools to optimise person-centred management and improve patient outcomes. Key causal pathways are identification of individual priorities and needs, individual care and treatment and integrated care. Communication of clinical uncertainty to patients is challenging and requires training and skill and the use of tools to support practice.

**Supplementary Information:**

The online version contains supplementary material available at 10.1186/s12904-021-00845-9.

## Introduction

People are living longer and increasingly die with multi-morbidities and frailty [1–4]. The last years of life for older people are often characterised by clinical uncertainty over recovery or continued deterioration leading to death. Clinical uncertainty is a challenging area of clinical care. It requires parallel planning and intervention to support recovery and to anticipate and plan for deterioration and dying [5]. Health and social care staff (practitioners) require expertise to communicate uncertainty with patients (including long term care residents) and families (including friends) and to manage multiple perspectives which are sometimes conflicting about treatment decisions, whilst ensuring that care is person-centred and aligned with the patient’s and family’s wishes and priorities [6, 7]. Poorly managed clinical uncertainty leads to poorer outcomes for patients and their family, including compromised quality of life [8].

Clinical uncertainty comprises multiple interlinked perspectives, such as the patient and practitioner [9–12] and levels such as the individual and service [13]. Studies have developed conceptual understanding for clinical care. Mishel [9–11] conceptualised uncertainty in illness as a clinical presentation that is ambiguous, complex with limited information and/or is unpredictable [10]. Goodman et al. (2015) pursued this understanding in care homes [13] identifying layers of treatment uncertainty arising for example from multi-morbidity, relationship uncertainty such as divergent priorities and service uncertainty such as workforce turnover. Etkind et al. [12] explored the views of patients to develop a typology of priorities in managing clinical uncertainty including level of engagement in decisions about care and treatment, tailored information to individual preferences and the time period an individual is focused.

Central to managing clinical uncertainty is person-centred care to align care and treatment with the patient’s and carer’s priorities and wishes. A person-centred tool is an instrument designed to support, facilitate or guide person-centred care or treatment and as such is a complex intervention. Examples of person-centred tools are patient-reported outcome measures to identify an individual’s priorities, symptoms or needs, or Advance Care Planning (ACP) tools to support patients in discussing and sharing their wishes for future care and treatment [14].

Managing clinical uncertainty for older adults with multimorbidity and frailty, is an important and complex area of clinical care, but no reviews have considered conceptually how using tools as complex interventions in clinical practice could support the management of clinical uncertainty and improve patient outcomes. This study aimed to develop a logic model by systematically identifying, appraising and synthesising the evidence on person-centred tools intended to support the management of clinical uncertainty for older people towards the end of life.

## Methods

This is a mixed methods systematic review using a results-based convergent design [15] to inform the logic model. The logic model is intended to: describe the components of person-centred tools; depict and conceptualise the causal pathways (how using them changes care and impacts on effect) and linkages with the intended outcomes and describe and understand the context in which this occurs [16].

We used the following methods to develop a process-based iterative logic model [17]. We started with a conceptual framework of clinical uncertainty in older people with multi-morbidity and frailty, informed by conceptual understanding from Mishel, Goodman et al. and Etkind et al. [9–13] (Additional file 1 - conceptual framework of clinical uncertainty). This conceptual framework underpinned our search strategy and initial data analysis to inform the development of the logic model [17]. From the conceptual framework we identified three domains of clinical uncertainty that we aimed to address: 1) comprehensive assessment targeting complexity and ambiguity, 2) communication of clinical uncertainty to patients and family targeting lack of information and 3) continuity of care (care planning, ACP and communicating within and across teams) targeting unpredictability. The logic model was iteratively reviewed and refined, informed by research project meetings, project steering group meetings and emerging review findings [17].

The systematic review followed Preferred Reporting Items for Systematic Reviews and Meta-Analyses (PRISMA) (Additional file 2 - PRISMA checklist). The protocol was registered on PROSPERO (CRD42018098566).

### Search strategy

The following databases were searched: IBSS (2000 - July 2018), Medline, PsycINFO and CINAHL, from year 2000 to December 2019 using a combination of MeSH terms and keyword terms. MeSH terms included “uncertainty” OR “disease progression” AND “chronic illness” OR “palliative care” OR terminal care AND “assessment” OR “outcome assessment” OR “care planning” OR “decision making” (Additional file 3 - full search strategy). The electronic search was supplemented by reference chaining and consulting experts in the field.

### Eligibility criteria

#### Participants

Adults aged 65 years and over, living with advanced or life-limiting condition(s), including cancer and chronic noncancer conditions, and nearing end of life. Nearing end of life encompassed: the last 1–2 years of life through to death, or using services or facilities associated with advanced disease e.g. receiving palliative care, residing in a care home. At least half of individual study populations needed to be within the above definitions [18].

#### Interventions/tools

The intervention comprised: (i) person-centred tools to inform clinical assessment of conditions, including outcome measures to assess physical symptoms and/or psychosocial concerns, tools to assess multi-dimensional clinical constructs, such as frailty and function, and those that support identification of person-centred goals; (ii) tools to support integrated care within and across settings, including, but not limited to, care and contingency planning tools, pathways and decision-support tools; and (iii) tools to support communication in advanced conditions between health and social care practitioners, the patient, and/or their families.

To maintain the focus of person-centred tools, we excluded assessments of individual symptoms e.g. pain, diagnostic, prognostication or risk assessment tools such as risk of mortality. Out of scope were models of care delivery, training interventions and systems of tool delivery e.g. telemonitoring or telehealth.

#### Control

All control groups and those with no controls.

#### Outcomes

All outcomes were included. We included carer and practitioner outcomes when these were included with patient outcomes.

#### Study design

We included published qualitative, quantitative and mixed-methods studies. Studies included development and evaluation of tools for clinical care e.g. cognitive interviews, studies that evaluated tools in clinical care, including randomised and non-randomised trials, process evaluations and quality improvement studies. Unpublished grey literature studies were ineligible as considered insufficiently robust evidence because, for example, not subject to peer review. Psychometric evaluations and tool development studies without use in clinical care were excluded. Reviews, clinical guidelines, case studies, opinion pieces, conference abstracts, theses and dissertations were also excluded.

#### Other limits

English language and human subjects.

### Study selection

All identified studies were managed using a reference management system (EndNote X9). One reviewer screened all titles and abstracts, and 10% of abstracts and titles were double-blind screened by a second reviewer (151 publications were double screened, 3 publications with divergence between assessors were reviewed by a third). Full text articles were reviewed by one reviewer and those with uncertain eligibility discussed with the full project team.

### Data extraction and quality assessment

Data extraction tables were developed, piloted and refined following discussion with all investigators. Fields extracted are detailed in Tables 1 and 2, and specific data to inform the logic model including implementation requirements, causal pathways, and acceptability and feasibility for routine clinical care.

**Table 1:** Characteristics of included studies

Lead author, country, quality assessment	Family of tools	Name and description of tool	Domain of uncertainty			Study design	Study population	Research question/aim	Primary outcomes/results/ conclusions
			Comprehensive assessment	Communication	Continuity of care				
Dunckley [19], 2005, UK, 0.45	POS	Palliative care Outcome Scale (POS) *The POS is a 10-item questionnaire covering physical and psychological symptoms; and spiritual, practical and emotional concerns.*	X		X	Qualitative study	Practitioners working in nursing homes or clinical hospices	To identify facilitators and barriers to implementing outcome measures.	Barriers to implementing POS included, a top-down decision-making approach, time-consuming tools, limited resources for data analysis and a lack of practitioner knowledge of the importance of using tools. Facilitators to successful implementation included practitioners being involved in implementation decisions and using a tool that can be adapted to clinical practice and organisational needs.
Tavares [20], 2017, Brazil, 0.7			X		X	Observational study	Patients admitted to a specialist palliative care unit. Mean age 77.5 years.	To implement the POS in a specialist palliative care inpatient unit in daily practice.	POS is feasible to implement and improves quality of care. Pain was particularly improved between timepoints.
Kane [21], 2018, UK, 0.6 Kane [22], 2017, UK,0.9		Integrated Palliative care Outcome Scale (IPOS) *The IPOS has 10 questions with two open questions covering patients’ main concerns and symptoms, respectively, and a five-point Likert scale (0–4) accompanying common symptoms, patient and family distress, existential well-being, sharing feelings with family, information available and practical concerns.*	X	X	X	A parallel, mixed methods embedded study	Advanced chronic heart failure patients in a nurse-led chronic heart failure disease management clinic. Mean age 75 years. Plus 4 nurses.	To examine the feasibility and acceptability of using a patient reported outcome measure and its potential to influence patient perceptions of care.	IPOS was feasible and acceptable to patients and practitioners for use in clinical care and research. IPOS also allowed patients to become more engaged in their clinical care and highlight their unmet needs.
Ellis-Smith [23], 2017, UK, 0.85		Integrated Palliative care Outcome Scale for Dementia (IPOS-Dem) *A 28-item questionnaire with all questions, apart from the first, rated on a 5-point scale.*	X	X	X	A multi-method qualitative study	Care home residents with dementia, family members, care home practitioners, GPs and district nurses.	To examine the content validity, acceptability and comprehension of IPOS-Dem for routine use in long-term care settings for people with dementia and to refine the tool.	IPOS-Dem is a comprehensive and acceptable way to detect symptoms and problems for those with dementia. It is also acceptable as a carer-reported measure. Refinements have been made to maximise caregiver expertise.
Ellis-Smith [24], 2018, UK, 0.9						A qualitative study with an embedded quantitative component	Care home residents with dementia, family members, care home practitioners, GPs and district nurses.	To explore the mechanisms of action, feasibility, acceptability and implementation requirements of a the IPOS-Dem	Key mechanisms of action were identified, and a theoretical model was developed. IPOS-Dem was shown to be acceptable and feasible. Assessment and management of symptoms and concerns is supported by IPOS-Dem.
Salisbury [25], 2018, UK, 0.86 Mann [26], 2019, UK, 0.85 Thorn [27] 2020, UK, 0.85	3D Approach	3D Approach *Replaces disease specific reviews of each health condition with one 6-montly comprehensive multidisciplinary review, including medication review.*	X	X	X	A pragmatic cluster-randomised controlled trial	Patients of participating GP surgeries with at least 3 chronic conditions. Mean age 71 years.	To implement, at scale, a new approach to managing patients with multimorbidity in primary care and to assess its effectiveness.	The 3D intervention did not improve patients’ quality of life. Both implementation and intervention failure were cited as reasons for failure. Cost effectiveness was equivocal.
Forbat [28], 2019, Australia, 0.73 Liu [29], 2020, Australia, 0.93		Palliative care needs rounds *Needs rounds are monthly clinical meetings that are conducted at the care facility that integrate a specialist palliative care perspective into nursing home care.*	X		X	A prospective stepped-wedge cluster randomised control trial	Care home residents. Mean age 85 years. Care home practitioners interviewed.	To determine whether a model of care providing specialist palliative care in care homes, called Specialist Palliative Care Needs Rounds, could reduce length of stay in hospital.	The primary outcome was length of stay in acute care. Secondary outcomes included number and cost of hospitalisations. Palliative care needs rounds reduced the number of hospitalisations and length of stay.
Forbat [30], 2018, Australia, 0.5		Development of checklist				A grounded theory ethnography		To describe the activities, thought processes and activities of practitioners that are generated within and from needs rounds. To develop a model that explains what occurs in needs rounds and distil checklist from this. To finalise the checklist.	The checklist was suitable to support the integration of specialist palliative care into residential care.
Waller [31], 2012, Canada, 0.91	Needs Assessment Tool	Needs Assessment Tool: Progressive Disease Cancer (NAT: PD-C) *One-page practitioner completed questionnaire*	X			Interrupted time series trial	Advanced cancer patients recruited from medical oncology, radiation oncology, and haematology outpatient clinics with an average age of 67 years.	To assess the impact of the systematic and ongoing use of the Guidelines and NAT: PD-C on patient outcomes including level of need, quality of life, anxiety, and depression.	The NAT:PD-C reduces health system and information needs, and patient care and support needs.
Janssen [32], 2019, The Netherlands, 0.73		Needs Assessment Tool: Progressive Disease Heart Failure (NAT: PD-HF) *One-page practitioner completed questionnaire*	X			Mixed methods	Outpatients with diagnosis of chronic heart failure. Average age 84.4 (SD: 7.7) years.	To translate and study the feasibility and acceptability of the NAT:PD-HF.	The NAT:PD-HF identified palliative care needs in all participants, and triggered action to address these in half. Palliative care communication skills training is required when implementing this tool.
Actcherberg [33], 2001, The Netherlands, 0.86	Resident Assessment Instrument	Resident Assessment Instrument (RAI) *The RAI consists of a structured screening questionnaire [the Minimum Data Set (MDS)], an algorithm that links the information from the MDS to certain important problem areas, and triggers protocols for these problem areas if required.*	X		X	Non randomised controlled trial	Residents admitted for long term care in a somatic ward. Average age 78.6 years	Does the implementation of the RAI method improve the quality of the co-ordination of care in Dutch nursing homes?	Improvements in case history, care plan, end of shift reporting, communication, patient allocation and patient report in the RAI group. RAI has the potential to improve the quality of co-ordination of care in nursing homes
Gestsdottir [34], 2015, Iceland, 0.91		InterRAI Palliative Care *The InterRAI PC is divided into 16 domains: demographics, health conditions, oral and nutritional status, skin condition, cognition, communication, mood and behaviour, psychosocial wellbeing, physical functioning, urinary and bowel continence, medications, treatments and procedures, responsibility/directives, social relationships, discharge or death, and assessment information.*	X			Longitudinal	Patients using the services of the palliative consultation team and hospital general and palliative care units	To assess the symptoms and functional status of patients at the point of admission to specialised palliative care in Iceland and to investigate whether symptoms and functional status change over time. Also, to examine the difference in symptoms and functional status between care settings. A secondary aim was to participate in the development of interRAI PC assessment tool	Symptom burden and functional loss were significantly experienced by patients from admission to discharge or death. Symptoms indicating progressive deterioration also increased in frequency and severity. Physical and cognitive function decreased at all levels. Inpatients had more symptoms and experienced more functional decline than home-care patients. The interRAI PC version 8 supported capture of important clinical information and monitoring changes over time.
Hill [35], 2002, New Zealand, 0.6	The Missoula-VITAS Quality of Life Index (MVQOLI)	The Missoula-VITAS Quality of Life Index (MVQOLI) *The Missoula-VITAS Quality of Life Index (MVQOLI) is a 25-item patient-centred index that weights each of five QOL dimensions by its importance to the respondent.*	X			A pre-test/post-test quasi-experimental design	72 hospice patients and 10 nursing practitioners. Ages ranging from 20 to 89 years old.	To examine the concept and measurement of quality of life (QOL) in terminally ill patients and how QOL can be improved within a hospice setting	Providing nurses with access to information on the patient’s QOL perspective better prepares them to meet the patient’s QOL needs. This results in clinically significant improvements to patient QOL.
Schwartz [36], 2005, USA, 0.77		Missoula-VITAS Quality of Life Index - Revised (MVQOLI-R) *As above, without the weighting.*	X		X	Psychometric evaluation and intervention study	End-stage renal disease patients and hospice, or long-term care facility, patients. Mean age 66.3 years.	To evaluate the MVQOLI-R from both psychometric and clinimetric perspectives.	The MVQOLI-R has clinical utility as a patient QOL assessment tool and may support communication between patients and clinicians.
Rockwood [37], 2000, Canada, 0.79	Comprehensive Geriatric Assessment	CGA and Goal Attainment Scale (GAS) *Unspecified group of tools used together in CGA.* *GAS is used to record patient goals and the achievement of those goals.*	X		X	Randomized, controlled, single-blinded trial	Frail patients living in a rural community. Mean ages 82.2 and 81.4 years.	Testing of the CGA in the common, but constrained, environment of frail older patients without nearby access to specialized care.	Intervention group more likely to achieve their goals. No change or difference in function, QOL, survival or time to institutionalisation.
Parlevliet [38], 2012, The Netherlands 0.86		CGA: *Comprising of: Charlson’s comorbidity index; Activities of Daily Living; Instrumental Activities of Daily Living; MMSE; SDGS; SNAQ; VAS; EuroQol-6D; IQCOD-SF; NPI-q; CAM; EDIZ; De Jong-Gierveldschaal*	X			Cross sectional comparative and feasibility study	Patients with end stage renal disease aged 65 years or above, either receiving peritoneal dialysis or haemodialysis in hospitals with dialysis facilities	To perform a systematic CGA to investigate somatic, psychological, functional and social function in a group of older dialysis patients. Secondly, we aimed to place our findings in a broader perspective by comparing our group to a population of elderly cancer patients who likewise suffered from an end-stage chronic progressive disease. Finally, we asked the multidisciplinary team for their opinion on the feasibility of the systematic CGA and the relevance of its outcome.	Geriatric conditions were highly prevalent among elderly dialysis patients and prevalence’s were comparable in both intervention and control populations. The CGA was feasible for use of recognition of conditions and overburdened carers.
Basic [39], 2002, Australia 0.86		CGA *Comprising of: Activities of Daily Living; Instrumental Activities of Daily Living; MMSE; GDS; SSI; Waterlow Risk Assessment Scale*	X			Observational study	Older people presenting to the emergency department who were considered at high risk of admission but who were not severely ill. Mean age 79.4 years	To evaluate the ability of the nurse to assess high risk elderly patients comprehensively. A secondary aim was to explore patient characteristics associated with referral to community aged care services from the emergency department.	A single nurse working in a busy emergency department can successfully identify patients with increased care needs, and direct high-risk patients to existing services.
Mariano [40], 2015, Canada 0.73	Geriatric assessment	Geriatric assessment (unspecified)	X			Pilot study	Cancer patients. Mean age 77 years. Hospitals	To evaluate the feasibility of GA in this frail, historically difficult-to-study population. Secondary objectives were to describe the level of deficits detected on GA, to assess whether hospital-based clinicians recognized and addressed these deficits, and to describe hospital-based outcomes including length of stay, discharge disposition, and 30-day readmission rates	GA was feasible in this population. Hospitalized older cancer patients experience more functional and psychosocial issues. Clinical recognition and management of these issues was poor. GA tools can be used to inform guide referrals to appropriate services.
Jadczak [41], 2017, Australia 0.59		Geriatric Assessment *Comprising of: FRAIL screen; CCI; SF-36; TMT; MNA-SF; RCS; Lawton IADL*	X			Observational study	Patients from a Geriatric Evaluation and Management Unit (GEMU) screened pre-frail or frail on the FRAIL Screen. Mean age 85.37 years.	To determine the feasibility of standardised geriatric assessments and standard physical exercises in hospitalised pre-frail and frail older adults	The FRAIL Screen, MNA-SF, Rapid Cognitive Screen, Lawton iADL and the physical exercises were deemed to be feasible with only minor comprehension, execution and safety issues. The TMT was not considered to be feasible and the SF-36 should be replaced by its shorter form, the SF-12.
Pepersack [42], 2008, Belgium, 0.67		Minimum Geriatric Screening Tools (MGST) *Battery of tools including: ADL; IADL; CSDD; Socios Scale; MUST; pain indicators; ISAR*	X			Prospective observational survey	Patients attending an acute geriatric unit, mean age 83.3 years.	The aims of this project were: 1) to assess the feasibility of a MGST within the teams of Belgian geriatric units; 2) to assess the efficacy of a MGST on the detection rate of the geriatric problems; and 3) to analyse quality variables within the data collected.	MGST leads to better assessment of geriatric domains (functional, continence, cognition, depression, nutrition, pain, social), apart from falls.
Cheang [43], 2014, Australia, 0.72	Advance Care Planning	ACP screening interviews *Guided interview*			X	Cross-sectional	Patients ages 80 years or over, who have been admitted for at least 48 h to an adult medical/surgical ward	To assess the prevalence of advanced care documents and documented medical orders regarding end-of-life care in the medical record of elderly inpatients and to explore the feasibility and acceptability of an advanced care planning screening interview.	Advance Care Directives and correct documentation of suitable decision-maker were uncommon in the medical records. The ACP screening interview appears feasible and acceptable and may be a useful tool for identifying suitable decision-maker and patients willingness to discuss ACP further.
Silvester [44] 2013, Australia 0.68		Advance Care Plan *Two-sided questionnaire asking about values and beliefs, unacceptable health condition, specific treatments wanted and unwanted.*			X	Audit of pre-existing documentation and pilot study	No patients recruited.	The development of the aged care specific Advance Care Plan template, the pre-implementation quality of ACP documents and the performance of the newly developed Advance Care Plan template	Standardised procedures and documentation are needed to improve the quality of processes, documents and outcomes of ACP.
Miller [45], 2019, Australia 0.75		Advance Care Planning *GP completes referral to GPN including health and social information. GPN conducts ACP discussion using an Advance Care Planning workbook and Advance Care Directive template was used to guide discussions and to record the patient’s wishes if required.*			X	Qualitative interviews	Patients of participating GP surgeries. Mean age 81 years.	To understand how patients experienced involvement in advanced care planning in the general practice setting when common barriers to uptake were addressed and what impact this has on patients and their families.	GPNs are able to hold ACP conversations with patients when provided with training and support. GPNs involvement in these conversations can benefit patients. Some patients may feel uncomfortable communicating results of ACP conversations with family.
Sudore [46], 2013, USA 0.9		Advance Care Planning Engagement survey *Survey with two sections containing 31 items in ‘process measures’ and 18 items in ‘action measures’.*			X	Development and psychometric evaluation	Patients recruited from hospitals, outpatient clinics and nursing homes. Mean age 69.3 years.	To develop and validate a survey designed to quantify the process of behaviour change in the advance care planning process.	The Advance Care Planning Engagement Survey measuring behaviour change and multiple advance care planning actions demonstrated good reliability and validity.
Bristowe [47], 2015, UK 0.85	The AMBER Care Bundle	The AMBER Care Bundle *This intervention* *has an algorithmic approach and is intended to encourage the clinical team to develop and document a clear medical plan and consider anticipated outcomes and resuscitation and escalation status; this is revisited daily.*		X	X	Mixed methods observational study	Patients in the acute hospital setting who are deteriorating, clinically unstable, with limited reversibility and at risk of dying in the next 1–2 months. Mean age 77 years.	Aims to examine the experience of care supported by the AMBER care bundle compared to standard care in the context of clinical uncertainty, deterioration and limited reversibility	Patients in the intervention group appeared to have higher awareness of prognosis. This does not translate to better quality communication and information was judged less easy to understand.
Koffman [48], 2019, UK, 0.82						Randomised controlled trial	Hospital inpatients. 38.5% were aged 60–79 years old, 46.2% were aged over 80 years old.	To investigate the feasibility of a cluster RCT of the AMBER care bundle.	The cluster RCT was feasible. However, optimal recruitment was prevented by impracticalities in the fundamental issues in operationalising the intervention’s eligibility criteria.
McMillan [49], 2011, USA, 0.86	Tools used together as a package	Patient instruments Palliative Performance Scale (PPS) Memorial Symptom Assessment Scale-Revised (MSAS) Hospice Quality of Life Index-14 (HQLI-14) Instruments for Both Patients and Caregivers Center for Epidemiological Study-Depression Scale (CES-D) Spiritual Needs Inventory Short Portable Mental Status Questionnaire	X			Clinical trial	Patients newly admitted to hospice care and their family caregivers. Patient mean age 72.66 years, caregiver mean age of 65.37 years.	To determine the efficacy of providing systematic feedback from standardized assessment tools for hospice patients and caregivers in improving hospice outcomes compared to the usual clinical practice	Depression scores were improved in the intervention group. Standard care received was so good that the overall quality of life improved as a result. This prevented improvement in other variables.
Gilbert [50], 2012, Canada, 0.55		Edmonton Symptom Assessment Symptom (ESAS), Palliative Performance Scale and Advance Care Plan	X		X	Mixed methods quality improvement	Cancer patients receiving community palliative care	The project involved 1) implementation of the ESAS for symptom screening, 2) use of “rapid-cycle change” quality improvement processes to improve screening and symptom management, and 3) improvements in integration and access to palliative care services.	The Provincial Palliative Care Integration Project demonstrated that by using rapid-cycle change and collaborative approaches, symptom screening and responses can be improved. Improvements can occur in the long and short term but require changes in system design and changes in clinical practice culture.
Mercandante [51], 2019, Italy, 0.82		Patient Dyspnea Goal, Patient Dyspnea Goal Response and Patient Global Impression *Patient Dyspnea Goal is an assessment tool to tailor symptom management, providing a therapeutic ‘target’. Patient Dyspnea Goal Response is the achievement of the goal. Global impression is global rating-of-change scale that assesses patients’ subjective response based on the individual feeling of improvement or deterioration.*	X		X	Secondary analysis	Advanced cancer patients admitted to palliative care units. Mean age 68.2 years.	To characterize the Patient Dyspnea Goal and Patient Dyspnea Goal Response, and Patients Global Impression after 1 week of a comprehensive symptom management. The secondary aim was to find possible factors influencing the clinical responses assessed as Patient Dyspnea Goal Response and Patient Global Impression.	Patient Dyspnea Goal Response and Patient Global Impression seem to be relevant for evaluating the effects of a comprehensive management of symptoms, assisting decision making process.
Cox [52], 2011, UK, 0.5		Edmonton Symptom Assessment Scale (ESAS) and the Euro-QoL (EQ-5D) *Technologies HealthHUB (held by patients) and CareHUB (held by clinicians) used as prompts to complete ESAS and EQ-5D questionnaires to assess symptoms and QoL respectively.*	X		X	Mixed methods	Hospice patients with a diagnosis of lung cancer	This study had two aims: [1] to test and evaluate the support provided to patients by the computerized assessment tool and [2] to determine the clinical acceptability of the technology in a palliative care setting.	Clinicians acknowledged patient and practice benefits of computerised patient assessment but highlighted the importance of clinical intuition over standardised assessment. While clinicians were positive about palliative care patients participating in research, they did indicate concerns around age and potential for rapid deterioration. The contribution of e-technology needs to be prompted, particularly in its potential to improve patient outcomes and experience, to encourage acceptance of its use in palliative care.
Hockley [53], 2010, UK, 0.82		Liverpool Care Pathway and Gold standards framework		X	X	Evaluation	Nursing home residents aged 66–103 years. 51% of residents had 3 or more diagnoses.	Using tools to help improve end-of-life care in care homes	There was a highly statistically significant increase in use of Do Not Attempt Resuscitation (DNAR) documentation, advance care planning and use of the LCP. An apparent reduction in unnecessary hospital admissions and a reduction in hospital deaths post-study were also found.
Jennings [54], 2016, USA, 0.95		Physician Orders for Life-Sustaining Treatment (POLST) *Legal document indicating preferences for life sustaining treatment*			X	Observational study	Residents in nursing facilities with a mean age of 78 years.	To evaluate the use of POLST among California nursing home residents, including variation by resident characteristics and by nursing home facility.	State-wide nursing home data show broad uptake of POLST in California without racial disparity. However, variation in POLST completion among nursing homes indicates potential areas for quality improvement.
Krumm [55], 2014, Germany, 0.8		Minimal Documentation system for Palliative Care (MIDOS) *One-page symptom assessment tool*	X		X	Qualitative multiple-unit study	Nurses and care assistants from specialist dementia units	To describe health professionals’ experiences of assessing the symptoms of people with dementia using a cancer-patient-oriented symptom-assessment tool from a palliative care context	The MIDOS tool was perceived as a helpful and valuable. Practitioners expressed some concerns regarding the subjective nature of perceiving symptoms and clinical decision making. The use of tools such as this has the potential to enhance the quality of palliative care in dementia care.
Landi [56], 2001, Italy, 0.93		Minimum Data Set for Home Care (MDS-HC)	X		X	Single blind randomized controlled trial	Older people living in the community receiving home care services	To test the effectiveness—in standardized home care programmes with case management—of a new, internationally validated assessment instrument, the MDS-HC	The intervention group used more at home services, were sent to hospital later and less often following assessment using MDS-HC assessment, therefore, reducing costs. MDS-HC also indicated improvements in physical and cognitive function in the intervention group.
Ratner [57], 2001, USA, 0.55		The Kitchen Table Discussion *Formally structured social work visits at patients’ homes to discuss end-of-life issues, with communication of results to home health nurses and attending physicians.*			X	Case series	Patients with a serious or life-threatening illness with a life expectancy of less than 2 years receiving home care. 75% aged 65 years and older.	To determine whether home health agency patients’ preferences to die at home can be honoured following a structured, professionally facilitated advance-care planning (ACP) process provided in the home.	Patients were willing to take part in ACP discussions at home. Most patients preferred to die at home. Facilitating ACP among such patients and their families was associated with end-of-life care at home. Use of hospice services was common following ACP in this population.
Schamp [58], 2006, USA, 0.68		Pathways Tool *A documentation tool that captures both present and advance directives in a framework of “pathways,” blending goals of care with typical procedure-oriented directives.*			X	Pre and post observational study design	Elderly, frail and medically complex population with an average of 8 chronic medical conditions living in the community. More than 133, of the 160 patients, were over the age of 65 years.	To determine the effect of using the Pathways Tool upon the rates of completion of health care wishes and whether the distinction of “present” versus “advance” directives might be associated with differing qualitative choices expressed	The Pathways Tool was associated with increased completion of health care wishes, preferences toward less invasive levels of care at life’s end, and increased compliance with participants’ wishes and deaths at home.
Zafirau [59], 2012, USA, 0.64		Resident Change in Condition Assessment/Transfer Form *The form provides background information on patient’s health history and other information helpful and necessary for receiving hospitals. It also records the presence of advanced directives. If a DNR order exists, a copy is attached directly to the form.*			X	Pre and Post test intervention evaluation	Patients in long term care facilities transferring to the emergency department, mean ages 72.8 and 76 years.	To test the efficacy of a standardized form used during transfers between long-term care facilities and the acute care setting	Communication between LTCFs of advanced directives was improved by use of the standardised transfer form. The form may also have increased admissions to the palliative care unit.
McGlinchey [60], 2019, UK, 0.8		Serious Illness Conversation Guide *Guide to support clinician’s communication with patients regarding current and future care and to promote shared decision making*		X	X	Stage 1: Nominal Group Technique Stage 2: Cognitive Interviews Stage 3: Stakeholder review and consensus	Stage 1: Medical oncologists, palliative care and communication skills experts. Stage 2: Lay representatives Stage 3: Stakeholders made up of lay members and health service practitioners and researchers.	To explore the ‘face validity’, applicability and relevance of the clinical tool, the Serious Illness Conversation Guide, to explore whether adaptations were required for the UK before its use in the pilot.	Interviews indicate acceptance from practitioners with some considerations. Use of the guide has the potential to benefit patients, facilitating a ‘person-centred’ approach to these important conversations, and to provide a framework to promote shared decision making and care planning.
Mills [61] 2018, Australia, 0.55		Goals-of-Care form *A one-page document used to guide and record discussions between clinicians and patients around care preferences.*		X	X	A prospective mixed methods study	108 forms were available from hospital inpatients. Median age 91 years. 16 doctors were interviewed.	To evaluate the utility to doctors of a form specifically designed to guide and document Goals of Care discussions at point of care. A secondary aim was to collect data on the length of GOC conversations and documentation.	Having a Goals-of-Care form in emergency medicine is supported. However, the ideal contents of the form were not determined.
Bouvette [62], 2002, Canada, 0.4		Pain and Symptom Assessment Record (PSAR) *Two-sided questionnaire*.	X		X	Mixed methods	Palliative care patients in acute care institutions and community palliative care and oncology services, such as hospices and nursing agencies	To determine the feasibility of implementing the Pain and Symptom Assessment Record (PSAR) to assess the pain and symptoms of palliative care patients in a variety of settings	Based on the results from this study, the tool has been modified and is currently utilized in a variety of settings.

Quality rating: < 0.60 = low; ≥0.60–0.79 = moderate; ≥0.80 = high

*ACP* Advance Care Plan (or planning), *ADL* Activities of Daily Living, *CAM* Confusion Assessment Method, *CGA* Comprehensive Geriatric Assessment, *CCI* Charlson Comorbidity Index, *CES-D* Center for Epidemiological Study-Depression Scale, *CHF* Chronic heart failure, *CSDD* Cornell Scale for Depression in Dementia, *DNAR* Do Not Attempt Resuscitation, *EDIZ* Experienced Burden of Informal Care, *EQ-5D* EuroQol-5D, *ESAS* Edmonton Symptom Assessment Symptom, *FRAIL screen* Fatigue, Resistance, Ambulation, Illness and Loss of weight screen, *GA* Geriatric assessment, *GAS* Geriatric Attainment Scale, *GDS* Geriatric Depression Scale, *GP* general practitioner, *GOC* Goals Of Care, *GPN* general practitioner nurse, *HQLI-14* Hospice Quality of Life Index-14, *IQCOD-SF* Informant Questionnaire Cognitive Decline – Short Form, *InterRAI PC* Residents Assessment Instrument - Palliative care, *IPOS* Integrated Palliative care Outcome Scale, *IPOS-Dem* Integrated Palliative care Outcome Scale for Dementia, *ISAR* Identification of Seniors at Risk, *(Lawton) IADL* (Lawton) Instrumental Activities of Daily Living, *LCP* Liverpool Care Pathway, *LTCF* Long Term Care Facility, *MDS-HC* Minimum Data Set for Home Care, *MGST* Minimum Geriatric Screening tool, *MIDOS* Minimal Documentation system for Palliative Care, *MMSE* Mini Mental State Examination, *MNA-SF* Mini Nutritional assessment – short form, *MSAS* Memorial Symptom Assessment Scale-Revised, *MUST* Malnutrition Universal Screening Tool, *MVQOLI (−R)* The Missoula-VITAS Quality of Life Index (−Revised), *NAT: PD-C* Needs Assessment Tool: Progressive Disease – Cancer, *NAT: PD-HF* Needs Assessment Tool: Progressive Disease – Heart Failure, *NPI-q* Neuropsychiatric Inventory Questionnaire, *POLST* Physician Orders for Life-Sustaining Treatment, *POS* Palliative care Outcome Scale, *PPS* Palliative Performance Scale, *PROM* Patient reported outcome measure, *PSAR* Pain and Symptom Assessment Record, *QOL* Quality of Life, *RCS* Rapid Cognitive Screen, *RCT* Randomised Controlled Trial, *SD* Standard deviation, *SF-36* Short Form survey, *SNAQ* Short Nutritional Assessment Questionnaire, *SSI* Social Support Instrument, *TMT* Trail Making Test, *VAS* Visual Analogue Scale

**Table 2:** Evidence of effectiveness

First author (country), study design and quality rating*	N	Tool	Domain of uncertainty			Outcome measured and results	Results and Interpretation
			Comprehensive Assessment	Communication	Continuity of care		
QUALITY OF LIFE							
Quality of life							
Hill, 2002 [35] New Zealand A pre-test/post-test quasi-experimental design 0.54	*N* = 72	Missoula-VITAS Quality of Life Index (MVQOLI)	x			*MVQOLI* - Overall: mean (SD) Control T1: 24.11 (33.70) Control T2: 35.00 (40.10) (ns) Intervention T1: 30.88 (41.88) Intervention T2: 47.41 (39.22) (*p* < 0.001) Between group, reported not significant	No effect between intervention and control group Within group improvement in intervention
McMillan, 2011 [49] USA RCT 0.86	*N* = 709	Package of tools with feedback of results to care team	x			*HQLI* - Model term: Estimate (SE), *p*-value Intercept: 102.33 (1.07), *p* < 0.001 Group: 1.65 (1.30), *p* = 0.206 Time: 0.29 (0.08), *p* < 0.001 Group x time: 0.03 (0.12), *p* = 0.811	No effect between intervention and control group Within group improvement in intervention
Salisbury, 2018 [25] UK Cluster RCT 0.86	*N* = 1546	3D approach	x	x	x	*EQ-5D-5L* – unadjusted mean (SE) Intervention: 0.533 (0.012) Control: 0.504 (0.012) Adjusted difference in means (95% CI): 0.00 (− 0.02–0.02)	No effect between intervention and control
Waller, 2012 [31] Canada Interrupted time series trial 0.91	*N* = 114	Needs Assessment Tool: Progressive Disease-Cancer (NAT:PD-C)	x			*EORTC QLQ-C30* Mean quality of life score (0–100) 6 months pre and 6 months post intervention: T-3: 64.5 (*p* < 0.05), T-2: 61.2, T-1: 61.2 T0: 58.0, T1: 57.5, T2: 56.5, T3: 57.5	No effect
Quality of death and dying							
Liu, 2019 [29] Australia Stepped Wedge RCT 0.93	*N* = 1700	Palliative Care Needs Rounds Checklist	x		x	*QODD* – mean (SD) Intervention: 72.4 (13.0) Control: 69.1 (13.6) Treatment effect (95% CI): 8.1 (3.8–12.4)	Effective
Health status							
Rockwood, 2000 [37] Canada RCT 0.79	*N* = 182	CGA and Goal Attainment Scale (GAS)	x		x	*Clinician’s global assessment* - Proportion improved Intervention: 39/85 Control: 15/80 *p* = 0.001	Effective
Janssen, 2019 [32] The Netherlands Pre-test/post-test pilot study 0.73	*N* = 17	Dutch Needs Assessment Tool: Progressive Disease – Heart Failure (NAT:PD-HF)	x			*Health status (MLHFQ) at baseline and 4 months*: *p* = 0.04	Worsening effect
Symptom control							
Tavares, 2017 [20] Brazil Observational study 0.7	*N* = 317	Palliative Outcome Scale/Palliative Outcome Scale-Symptoms (POS/POS-S)	x		x	*POS* – Number and percentage of patients scoring moderate or high (≥2) at T0 with any improvement at T1 Pain: *n* = 10/11 (91%), *p* = 0.01 Other symptoms: *n* = 7/11 (64%), *p* = 0.03	Effective
						*POS –* Number and percentage of patients scoring moderate or high (≥2) at T0 with any improvement at T1 Anxiety: *n* = 5/17 (29%), *p* = 0.35 Family anxiety: *n* = 3/20 (15%), *p* = 0.73 Information: *n* = 1/1 (100%) Support: *n* = 1/1 (100%) Depression: *n* = 2/5 (40%), *p* = 0.18 Self-worth: *n* = 1/4 (25%), p = 1.00 Time wasted: *n* = 3/3 (100%), *p* = 0.10 Personal affairs: *n* = 0/2 (0%), *p* = 1.00	No effect
						*Modified POS-S* - Percentage of patients scoring moderate or high (≥2) at T0 with any improvement at T1 Pain: *n* = 45/51 (88%), *p* < 0.001 Shortness of breath: *n* = 42/50 (84%), *p* < 0.001 Poor appetite: *n* = 18/42 (42%), *p* = 0.02 Constipation: *n* = 24/31 (77%), *p* < 0.001 Mouth problems: n = 17/25 (68%), *p* = 0.00 Drowsiness: *n* = 45/83 (54%), *p* < 0.001 Anxiety or agitation: *n* = 31/49 (63%), *p* < 0.001 Nausea/vomiting: *n* = 12/15 (80%), *p* = 0.00 Insomnia: *n* = 12/15 (80%), *p* = 0.01 Diarrhoea: n = 7/8 (88%), *p* = 0.01	Effective
Ellis-Smith, 2018 [24] UK Single arm mixed methods feasibility and process evaluation 0.9	*N* = 30	Integrated Palliative care Outcome Scale – Dementia (IPOS-Dem)	x	x	x	*IPOS-Dem* - Mean (SD) Baseline total score: 15.47 (10.51) Final time point total score: 15.82 (10.94)	No effect
Gestsdottir, 2015 [34] Iceland Prospective longitudinal 0.91	*N* = 81	Inter Resident Assessment Instrument - Palliative Care (InterRAI-PC)	x			*InterRAI-PC* - Mean rank T1, T2, T3, X^2^, p-value Fatigue 1.99, 1.93, 2.08, 3.783, *p* = 0.151 Pain frequency 1.95, 1.89, 2.16, 4.866, *p* = 0.088 Pain strength 1.91, 1.94, 2.15, 4.071, *p* = 0.131 Difficulty sleeping 2.02, 1.88, 2.10, 3.957, *p* = 0.138 Nausea 2.16, 1.92, 1.93, 6.7, *p* = 0.035 Constipation 2.03, 1.91, 2.06, 1.694, *p* = 0.429 Oedema 1.90, 2.04, 2.06, 4.825, *p* = 0.090 Change in usual sleeping patterns 2.07, 1.87, 2.05, 3.206, *p* = 0.201 Sadness 1.98, 1.92, 2.09, 2.341, *p* = 0.310 Reduced social interaction 1.98, 1.88, 2.14, 4.200, *p* = 0.122	No effect
						*InterRAI-PC* - Mean rank T1, T2, T3, X^2^, *p*-value Loss of appetite 1.96, 1.83, 2.21, 11.346, *p* = 0.003 Insufficient nutritional intake 1.93, 1.84, 2.23, 14.510, *p* = 0.001 Shortness of breath with exertion 1.96, 1.87, 2.16, 10.393, *p* = 0.006 Dry mouth 1.83, 1.99, 2.18, 12.797, *p* = 0.002	Worsening symptoms
Janssen, 2019 [32] The Netherlands Pre-test/post-test pilot study 0.73	*N* = 17	NAT:PD-HF	x			*Symptom distress (ESAS) score at baseline and 4 months*: *p* = 0.78	No effect
Illness burden							
Salisbury, 2018 [25] UK Cluster RCT 0.86	*N* = 1546	3D approach	x	x	x	*Self-rated health of good or better* - n/N (%) Intervention: 242/642 (38%) Control: 230/631 (36%) Adjusted odds ratio (95% CI): 0.845 (0.67–1.05)	No effect
						*Bayliss measure of illness burden* - Mean (SD) Intervention: 16.7 (11.6) Control: 18.4 (12.9) Adjusted beta-coefficient (95% CI): −0.64 (−1.54–0.27)	No effect
Needs							
Waller, 2012 [31] Canada Interrupted time series trial 0.91	*N* = 114	NAT: PD-C	x			*Supportive Care Needs Survey and spiritual domain of NAT: PD-C -* Percentage of people reporting at least one moderate or high need T0: 64%, T1: 61%, T2: 51%, T3: 52% (z = 1.73, *p* = 0.08)	No effect
Goal Attainment							
Rockwood, 2000 [37] Canada RCT 0.79	*N* = 182	CGA and GAS	x		x	*GAS at 3 months* Intervention: Total GAS $\overline{\boldsymbol{x}}$ = 46.4 ***±*** 5.9, Outcome GAS $\overline{\boldsymbol{x}}$ = 48.0 ***±*** 6.6 Control: Total GAS $\overline{\boldsymbol{x}}$ = 38.7 ***±*** 4.1, Outcome GAS $\overline{\boldsymbol{x}}$ = 40.8 ***±*** 5.6 *p* < 0.001	Effective
Psychological/spiritual wellbeing							
McMillan, 2011 [49] USA RCT 0.73	*N* = 709	Package of tools with feedback of results to care team	x			*CES-D* - Model term: Estimate (SE), *p*-value Intercept: 4.51 (0.11), *p* < 0.001 Group: 0.01 (0.13), *p* = 0.929 Time: −0.02 (0.01), *p* = 0.23 Group x time: − 0.03 (0.01), *p* = 0.027	Effective
						*MSAS distress* - Model term: Estimate (SE), *p*-value Intercept: 1.99 (0.06), *p* < 0.001 Group: −0.08 (0.07), *p* = 0.238 Time: − 0.01 (0.01), *p* = 0.628 Group x time: 0 (0.01), *p* = 0.991	No effect
						*Spiritual Needs Inventory* - Model term: Estimate (SE), *p*-value Intercept: 1.67 (0.10), *p* < 0.001 Group: −0.23 (0.12), *p* = 0.062 Time: − 0.02 (0.09), *p* = 0.058 Group x time: 0.02 (0.01), *p* = 0.158	No effect
Salisbury, 2018 [25] UK Cluster RCT 0.86	*N* = 1546	3D approach	x	x	x	*Depression (HADS)* - Mean (SD) Intervention group: 6.1 (4.6) Control group: 6.8 (4.6) Adjusted beta coefficient (95% CI): − 0.01 (− 0.33–0.30)	No effect
						*Anxiety (HADS)* - Mean (SD) Intervention group: 5.8 (4.7) Control group: 6.3 (4.8) Adjusted beta coefficient (95% CI): −0.24 (− 0.57–0.08)	
Waller, 2012 [31] Canada Interrupted time series trial 0.91	*N* = 114	NAT:PD-C	x			*Clinical depression (HADS) -* Percentage of patients with score 11+ 6 months pre and 6 months post intervention: T-3 9.9, T-2 8.4 (*p* < 0.05), T-1 10.2, T0 13.5, T1 9.5, T2 10.9, T3 13.8	No effect
						*Clinical anxiety (HADS) -* Percentage of patients with score 11+ 6 months pre and 6 months post intervention: T-3 8.8, T-2 8.1, T-1 8.5, T0 9.2, T1 9.2, T2 13.5, T3 8.1	
FUNCTION							
Functional status/ADL							
Landi, 2001 [56] Italy RCT 0.93	*N* = 176	Minimum Data Set for Home Care (MDS-HC)	x		x	*Barthel Index* - Adjusted mean (SD) Intervention: 51.7 (36.1) Control: 46.3 (33.7) *p* = 0.05	Effective
						*IADL – Lawton Index* - Adjusted mean (SD) Intervention: 23.5 (5.9) Control: 21.9 (6.6) *p* = 0.4	No effect
Gestsdottir, 2015 [34] Iceland Prospective longitudinal 0.91	*N* = 81	InterRAI-PC	x			*Change in physical function (InterRAI-PC)* - Mean rank T1, T2, T3 X^2^, *p*-value Personal hygiene 1.62, 1.81, 2.57, 69.926, *p* = 0.001 Toilet use 1.71, 1.87, 2.42, 42.683, *p* = 0.001 Walking ability 1.71, 1.83, 2.46, 47.523, *p* = 0.001 Bed mobility 1.62, 1.83, 2.54, 66.953, *p* = 0.001 Eating 1.64, 1.81, 2.56, 73.345, *p* = 0.001 Use of urinary collection device 1.85, 1.98, 2.17, 10.950, *p* = 0.004 Bowel continence 1.83, 1.86, 2.30, 24.093, *p* = 0.001	Worsening effect
Janssen, 2019 [32] The Netherlands Pre-test/post-test pilot study 0.73	*N* = 17	NAT:PD-HF	x			*Performance status (AKPS) at baseline and 4 months:* *p* = 0.10	No effect
						*Care dependency (CDS): number of symptoms at baseline and 4 months:* *p* = 0.43	No effect
Cognitive function							
Landi, 2001 [56] Italy RCT 0.93	*N* = 176	MDS-HC	x		x	*MMSE* - Adjusted mean (SD) Intervention: 19.9 (8.9) Control: 19.2 (10.7) *p* = 0.03	Effective
Gestsdottir, 2015 [34] Iceland Prospective longitudinal 0.91	*N* = 81	InterRAI-PC	x			*Change in cognitive function (InterRAI-PC)* - Mean rank T1, T2, T3 X^2^, *p*-value Cognitive skills for daily decision making 1.71, 1.86, 2.41, 39.282, *p* = 0.001	Worsening effect
SATISFACTION/QUALITY OF CARE							
Patient-centred care							
Salisbury, 2018 [25] UK Cluster RCT 0.86	*N* = 1546	3D approach	x	x	x	*PACIC* – Mean (SD) Intervention group: 2.8 (1.0) Control group: 2.5 (0.9) Adjusted beta coefficient (95% CI): 0.29 (0.16–0.41)	Effective
						*CARE doctor* – Mean (SD) Intervention group: 40.2 (9.7) Control group: 37.5 (10.0) Adjusted beta coefficient (95% CI): 1.20 (0.28–2.13)	Effective
						*CARE nurse* – Mean (SD) Intervention group: 40.8 (8.9) Control group: 38.5 (9.5) Adjusted beta coefficient (95% CI): 1.11 (0.03–2.19)	Effective
						*Patients reporting that they almost always discuss the problems most important to them in managing their own health* – n/N (%) Intervention group: 256/612 (42%) Control group: 153/599 (26%) Adjusted odds ratio (95% CI): 1.85 (1.44–2.38)	Effective
						*Patients reporting that support and care is almost always joined up* - n/N (%) Intervention group: 257/614 (42%) Control group: 173/603 (29%) Adjusted odds ratio (95% CI): 1.48 (1.18–1.85)	Effective
						*Patients reporting being very satisfied with care* - n/N (%) Intervention group: 345/614 (56%) Control group: 236/608 (39%) Adjusted odds ratio (95% CI): 1.57 (1.19–2.08)	Effective
						*Patients reporting having a written care, health, or treatment plan* - n/N (%) Intervention group: 141/623 (23%) Control group: 91/623 (15%) Adjusted odds ratio (95% CI): 1.97 (1.32–2.95)	Effective
HEALTH SERVICE USE							
Hospital admission/readmission							
Landi, 2001 [56] Italy RCT 0.93	*N* = 176	MDS-HC	x		x	*Number of persons admitted at least once* Intervention: 14.8% (*n* = 13) Control: 26.1% (*n* = 23) Relative Risk: 0.49 (95% CI: 0.56–0.97)	Effective
						*Time to first hospital admission* Log rank *p* = 0.05	
Zafirau, 2012 [59] USA Pre-test/post-test 0.64	Pre-intervention *N* = 130 Post-intervention *N* = 117	Resident Change in Condition Assessment/Transfer Form			x	*Readmission within 30 days* Pre intervention: 28.2% Post intervention: 22.2% *p* = 0.280	No effect
						*Admissions to ICU, CCU, telemetry* Pre intervention: 34% Post intervention: 47% *p* = 0.053	
						*Treated and released from ER* (%) Pre intervention: 79% Post intervention: 32% *p* = 0.329	
Rockwood, 2000 [37] Canada RCT 0.79	*N* = 182	CGA and GAS	x		x	*Institution-free survival -*Days of institution-free survival Intervention: 340, SE = 9 Control: 342, SE = 8 Log rank = 0.661, *p* = 0.416	No effect
						*Proportion institutionalised* Intervention: 13/95 Control: 8/87 X^2^ = 0.634, *p* = 0.426	
Salisbury, 2018 [25] UK Cluster RCT 0.86	*N* = 1546	3D approach	x	x	x	*Hospital admissions* - Median (IQR) Intervention group: 0.0 (0.0–1.0) Control group: 0.0 (0.0–1.0) Adjusted incidence rate ratio (95% CI): 1.04 (0.84–1.30)	No effect
Hospital length of stay							
Forbat, 2019 [28] Australia Step-wedged RCT 0.73	*N* = 1700	Palliative Care Needs Round Checklist	x		x	*Length of hospital stay (days)* – Mean (SD) Intervention: 6.4 (8.3) Control: 6.9 (9.1) Treatment effect: − 0.22, 95% CI − 0.44—0.01, *p* = 0.038	Effective
Bristowe, 2015 [47] UK Comparative observational 0.85	*N* = 60	Amber Care Bundle		x	x	*Length of hospital stay (days)* – Mean (SD, median, range) Intervention: 20.3 (19.2, 14, 1–87) Comparison: 29.3 (20.4, 21, 6–70) *p* = 0.10	No effect
Landi, 2001 [56] Italy RCT 0.93	*N* = 176	MDS-HC	x		x	*Total number of hospital days* Intervention: 273 Control: 631 *p* = 0.40	No effect
						*Number of hospital days per user* – Mean (SD) Intervention: 21.0 (13.4) Control: 27.4 (26.9) *p* = 0.40	
						*Number of hospital days per admission* – Mean (SD) Intervention: 13.3 (7.9) Control 20.8. (14.8) *p* = 0.08	
Zafirau, 2012 [59] USA Pre-test/post-test 0.64	Pre-intervention. *N* = 130 Post-intervention *N* = 117	Resident Change in Condition Assessment/Transfer Form			x	*Length of hospital stay (days)* Pre-intervention: 5.77 Post-intervention: 6.79 *p* = 0.058	No effect
						*Length of hospital stay excluding hospice patients (days)* Pre-intervention: 5.8 Post-intervention: 6.3 *p* = 0.480	
Place of death							
Schamp, 2006 [58] USA Pre-test/post- interventional cohort 0.68	Pre-intervention deaths *N* = 33 Post-intervention deaths *N* = 49	Pathways tool			x	*Deaths at home* Before intervention: 24% After intervention: 65% *p* < 0.001	Effective
Bristowe, 2015 [47] UK Comparative observational 0.85	*N* = 79	Amber Care Bundle		x	x	*Place of death* Intervention: Home or home of relative or close friend: 20% Hospice: 20% Hospital: 51% Care home: 9% Comparison: Home or home of relative or close friend: 9% Hospice: 9% Hospital: 68% Care home: 14% X^2^ = 5.71, *p* = 0.126	No effect
Treatment/services received							
Rockwood, 2000 [37] Canada RCT 0.79	*N* = 182	CGA and GAS	x		x	*Proportion receiving pneumococcal inoculation* (%) Intervention: 10% (*n* = 8/81) Control: 1% (*n* = 1/74) *P* = 0.013	Effective
Zafirau, 2012 [59] USA Pre-test/post-test 0.64	Pre-intervention *N* = 130 Post-intervention *N* = 117	Resident Change in Condition Assessment/Transfer Form			x	*Admission to hospice* (%) Pre intervention: 1.5% Post intervention: 7.7% *P* = 0.015	Effective
						*Admitted to geropsychiatry* (%) Pre-intervention: 1.7% Post-intervention: 2.3% *p* = 0.136	No effect
						*Change in CPR, intubation, cardioversion performed* (%) Pre intervention: 12% Post intervention: 9% *p* = 0.460	
						*Feeding tube, surgery performed* (%) Pre intervention: 19% Post intervention:23% *p* = 0.290	
Landi, 2001 [56] Italy RCT 0.93	*N* = 176	MDS-HC	x		x	*Use of community services: Home help (hours/year/patient)* – Mean (SD) Intervention: 59.2, (18.0) Control: 14.7 (5.6) *p* = 0.02	Effective
						*Use of community services: Home nursing (hours/year/patient)* – Mean (SD) Intervention: 28.3 (5.1) Control: 22.9 (2.1) *p* = 0.30	No effect
						*Use of community services: Physiotherapist (hours/year/patient)* – Mean (SD) Intervention: 11.2 (2.1) Control: 10.2 (1.6) *p* = 0.70	
						*Use of community services – GP (home visits/year/patient)* – Mean (SD) Intervention: 9.8 (1.2) Control: 10.1 (1.3) *p* = 0.80	
Salisbury, 2018 [25] UK Cluster RCT 0.86	*N* = 1546	3D approach	x	x	x	*Nurse consultations* – Median (IQR) Intervention group: 6.0 (4.0–10.0) Control group: 4.0 (2.0–8.0) Adjusted incidence rate ratio (95% CI): 1.37 (1.17–1.61) *p* = 0.0001	Effective
		3D approach	x	x	x	*Primary care physician consultations* – Median (IQR) Intervention group: 10.0 (6.0–16.0) Control group: 8.0 (4.0–14.0) Adjusted incidence rate ratio (95% CI): 1.13 (1.02–1.25) *p* = 0.0209	
		3D approach	x	x	x	*High risk prescribing* – Median (IQR) Intervention group: 0.0 (0.0–1.0) Control group: 0.0 (0.0–1.0) Adjusted incidence rate ratio (95% CI): 1.04 (0.87–1.25) *p* = 0.680	No effect
		3D approach	x	x	x	*Hospital outpatient attendances* – Median (IQR) Intervention group: 3.0 (1.0–5.0) Control group: 2.0 (1.0–5.0) Adjusted incidence rate ratio (95% CI): 1.02 (0.92–1.14) *p* = 0.720	
Bristowe, 2015 [47] UK Comparative observational 0.85	*N* = 76	Amber Care Bundle		x	x	*Involvement of palliative care (%)* Intervention: 60% Comparison: 61% X^2^ = 0.001, *p* = 0.980	No effect
McMillan, 2011 [49] USA RCT 0.73	*N* = 709	Package of tools with feedback of results to care team	x			*Number of contacts (visits or calls) by members of interdisciplinary team* - Mean (SD) at T1, T2, T3 Nurse visits: 3.4 (1.4), 2.2 (1.4), 2.5 (1.7) Home Health Aide: 0.50 (1.1), 0.80 (1.4), 0.9 (1.5) Volunteer visits: 0.02 (0.15), 0.06 (0.31) 0.05 (0.23) Physician visits: 0.3 (0.5), 0.2 (0.4), 0.2 (0.4) Psychosocial visits: 1.2 (0.6), 0.5 (0.6), 0.6 (0.7) Chaplain visits: 0.1 (0.3), 0.2 (0.4), 0.2 (0.5) Advanced Registered Nurse Practitioner: 0.1 (0.4), 0.1 (0.3), 0.1, (0.3) No change over time within groups (*p* > 0.05), and not modified by intervention (*p* > 0.05).	No effect
Treatment burden/quality of disease management							
Salisbury, 2018 [25] UK Cluster RCT 0.86	*N* = 1546	3D approach	x	x	x	*Multimorbidity Treatment Burden Questionnaire* – Mean (SD) Intervention group: 12.9 (15.0) Control group: 15.0 (17.1) Adjusted beta coefficient (95% CI): −0.46 (−1.78–0.86)	No effect
						*Eight-item Morisky Medication Adherence* – Mean (SD) Intervention group: 6.7 (1.2) Control group: 6.6 (1.3) Adjusted beta coefficient (95% CI): 0.06 (− 0.05–0.17)	
						*Number of different drugs prescribed in past 3 months* – Median (SE) Intervention group: 11.0 (8.0–15.0) Control group: 11.0 (8.0–15.0) Adjusted incidence rate ratio (95% CI): 1.02 (0.97–1.06)	
						*Number of QOF indicators met (quality of disease management)* – Mean (SD) Intervention group: 84.3 (17.5) Control group: 85.6 (17.3) Adjusted beta coefficient (95% CI): 0.41 (−3.05–3.87)	
SURVIVAL							
Mortality/survival							
Landi, 2001 [56] Italy RCT 0.93	*N* = 176	MDS-HC	x		x	*One-year mortality* (%) Intervention: 30.5% Control: 29.5% RR = 1.05, 95% CI = 0.55–2.01	No difference in survival/mortality
Rockwood, 2000 [37] Canada RCT 0.79	*N* = 182	CGA and GAS	x		x	*12-month survival -* Proportion died Intervention: 13/95 Control: 7/87 X^2^ = 1.476, *p* = 0.224	No difference in survival/mortality
						*Survival time* Intervention $\overline{\boldsymbol{x}}$ = 320 days (SE = 6) Controls $\overline{\boldsymbol{x}}$ = 294 days (SE = 6) Log rank = 1.284, *p* = 0.257	No difference in survival/mortality
CARER OUTCOMES							
Janssen, 2019 [32] The Netherlands Pre-test/post-test pilot study 0.73	*N* = 17	NAT:PD-HF	x			*FACQ-PC at baseline and 4 months*: Caregiver strain: *p* = 0.10 Caregiver distress: *p* = 0.48 Positive caregiving appraisal: *p* = 0.53 Family wellbeing: *p* = 0.94	No effect
McMillan, 2011 [49] USA RCT 0.73	*N* = 709	Package of tools with feedback of results to care team	x			*Received support* - Model term: Estimate (SE), *p*-value Intercept: 3.67 (0.03), *p* < 0.001 Group: 0.02 (0.04), *p* = 0.618 Time: 0 (0), *p* = 0.964 Group x time: 0.01 (0), *p* = 0.228	No effect
						*CES-D* - Model term: Estimate (SE), p-value Intercept: 4.48 (0.10), *p* < 0.001 Group: −0.11 (0.12), *p* = 0.367 Time: −0.01 (0.01), *p* = 0.104 Group x time: − 0.01 (0.01), *p* = 0.574	
						*Spiritual needs inventory* - Model term: Estimate (SE), p-value Intercept: 1.21 (0.14), *p* < 0.001 Group: −0.08 (0.17), *p* = 0.637 Time: 0.01 (0.01), *p* = 0.271 Group x time: 0.02 (0.02), *p* = 0.138	
COSTS							
Forbat, 2019 [28] Australia Step-wedged RCT 0.73	*N* = 1700	Palliative Care Needs Rounds Checklist	x		x	*Overall annual net cost-saving across 12 sites:* A$1759, 011 (US$1.3 m; UK£0.98 m) Years 2017–2018	Cost effective
Landi, 2001 [56] Italy RCT 0.93	*N* = 176	MDS-HC	x		x	*Total* per capita *health care costs* Intervention: $837 Control: $1936 Years 1998/1999 *p* < 0.01	Cost effective
Thorn 2020 [27] UK Pragmatic cluster RCT 0.85	*N* = 1546	3D approach	x	x	x	*Adjusted QALYs over 15 months of follow-up* - Mean (SE) Intervention: 0.675 (0.006) Control: 0.668 (0.006) Years 2015–2016 Incremental difference (95% CI): 0.007 (−0.009–0.023)	Not cost-effective
						*Adjusted costs from the NHS/PSS perspective* - Mean (SE) Intervention: £6140 (333) Control: £6014 (343) Years 2015–2016 Incremental difference (95% CI): £126 (£-739-£991)	
						ICER: £18,499 Years 2015–2016 Net monetary benefit at £20,000 (95% CI): £10 (£-956-£977)	

*AKPS* Australia-modified Karnofsky Performance Status, *CARE* Consultant and relational empathy, *CES-D* Center for Epidemiological Study-Depression Scale, *EQ-5D-5L* EuroQol-5D 5 level, *EORTC QLQ-C30* European Organization for Research and Treatment of Cancer Quality of Life Questionnaire, *FACQ-PC* Family Appraisal of Caregiving Questionnaire, *HADS* Hospital anxiety and depression scale, *HQLI* Hospice quality of life index, *IADL* Instrumental activities of daily living, *ICER* Incremental cost-effectiveness ratio, *IQR* Interquartile range, *MMSE* Mini mental state examination, *MSAS* Memorial symptom assessment scale-revised, *NHS* National health service, *PACIC* Patient Assessment of Care for Chronic Conditions, *PSS* Personal social services, *QALY* Quality-adjusted life year, *QODD* Quality of death and dying, *QOF* Quality and outcomes framework, *RCT* Randomised controlled trial, *SD* Standard deviation, *SE* Standard error

We used QualSyst to appraise the quality of included studies [63]. One reviewer assessed the quality of each of the papers. We graded the quality of papers as strong (≥0.80), medium (≥0.60–0.79) and low (< 0.60) [64, 65]. A random 10% sample was assessed by a second reviewer. Scores that diverged by > 10% were discussed within the research team. For mixed methods study, we quality rated the dominant method that the study employed and gave the corresponding quality rating.

### Data analysis and data synthesis

We used a results-based convergent synthesis design [15] to incorporate disparate data from qualitative and quantitative studies, in order to understand the processes of using tools in clinical care and the outcomes on care, and used data triangulation to strengthen the findings. Qualitative and quantitative data were analysed and presented separately, and the findings integrated into figures [15]. Qualitative data of the included papers’ results sections and quotations were thematically analysed using an a priori coding tree. This was informed by our conceptual framework of clinical uncertainty [9–13], and a theoretical model of using a person-centred outcome measure to improve outcomes of care [24]. We inductively developed additional codes for data relevant to our aim, but not in our a priori coding tree. The codes were then inductively themed. Qualitative data analysis was conducted by three investigators and all analysis was discussed in research meetings with the research team. We conducted narrative synthesis of quantitative data.

Outcomes and intervention components were defined and categorised in accordance with Rohwer et al. [66], and our conceptual framework of clinical uncertainty [9–13]. Intervention components and causal pathways were examined and presented according to the domains of our conceptual framework of clinical uncertainty (comprehensive assessment, communication with patients and families, continuity of care). Similarly, for effectiveness studies, we examined and presented outcomes by the domains of clinical uncertainty that the tools targeted. Only studies that had a comparison group, and presented and analysed comparator data to examine effect on the stated outcome, were included in the narrative synthesis. As we did not have any a priori criteria for acceptability and feasibility, and recognised that these may be different dependent on tool and setting, we did not report quantitative data on acceptability and feasibility.

## Results

### Study selection

**Fig. 1:**
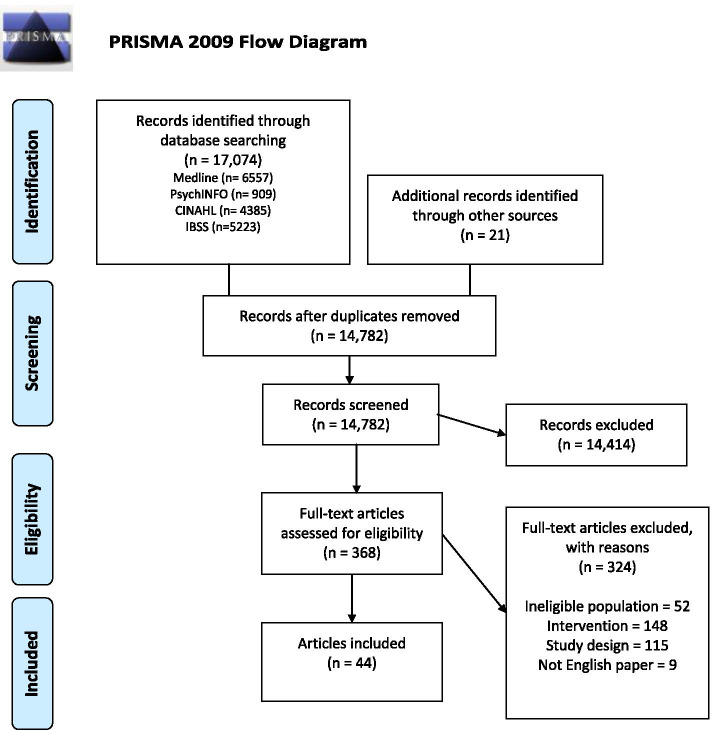
PRISMA flowchart

We identified 17,074 articles. Forty four articles met the eligibility criteria, reporting 40 studies (Fig. 1). After duplicates were removed, 14,782 articles were screened, including 21 articles retrieved from hand searching methods. From title and abstract screening, 368 articles proceeded to full text review. Studies were excluded at full text review due to ineligible population (*n* = 52), intervention (*n* = 148), study design (*n* = 115) and not written in English (*n* = 9).

### Study characteristics and participants

Most of the included studies were conducted in the UK (*n* = 11), Australia (*n* = 8) or USA (*n* = 8). Study settings included hospitals (*n* = 12), community (including patient home, care agencies and GP surgeries) (*n* = 12), specialist units (including geriatric, palliative care and disease specific) (*n* = 8), hospice (*n* = 5) and care homes with or without nursing (*n* = 6; *n* = 5). Twenty-one articles were assessed as high quality (14 quantitative [25, 26, 29, 31, 33, 34, 38, 39, 46, 48, 51, 53, 54, 56], 7 qualitative [22–24, 27, 47, 55, 60]), 15 as medium quality (12 quantitative [20, 28, 32, 36, 37, 40, 42–44, 49, 58, 59], 3 qualitative [21, 35, 45])and 8 as low quality (3 quantitative [41, 50, 61], 5 qualitative [19, 30, 52, 57, 62]) (Table 1).

The number of participants included in studies ranged from 13 to 289,753, with approximately 54% female participants. Participants’ average age was 77.4 years and ranged from 28 to 103 years old. Most participants were patients, four studies included family members/carers and 9 studies included practitioners.

Sixty-three tools were identified over the 40 studies (Table 1). The Palliative care Outcome Scale (POS), and versions of it, were reported in six publications [19–24]. Three articles were included reporting the 3D approach study [25–27] and two studies examined the Palliative Care Needs Rounds tool across three publications [28–30]. Four tools and/or versions were identified in multiple studies (POS *n* = 4; Needs Assessment Tool (NAT) *n* = 2; Resident Assessment Instrument (RAI) *n* = 2; Missoula-VITAS Quality of Life Index (MVQOLI) *n* = 2). Six studies included Comprehensive Geriatric Assessment (CGA) [37–39] or geriatric assessments [40–42], however two of these studies did not define specific tools [37, 40]. Five studies examined ACP, including one as a part of a package of tools [43–46, 50]. Two articles reported on two ‘packages of tools’, meaning more than a single tool was used [49, 50].

Comprehensive assessment was the domain most targeted (31 publications) and communication was the least targeted (8 publications) (Table 1).

### Causal pathways of tools used to manage clinical uncertainty

**Fig. 2:**
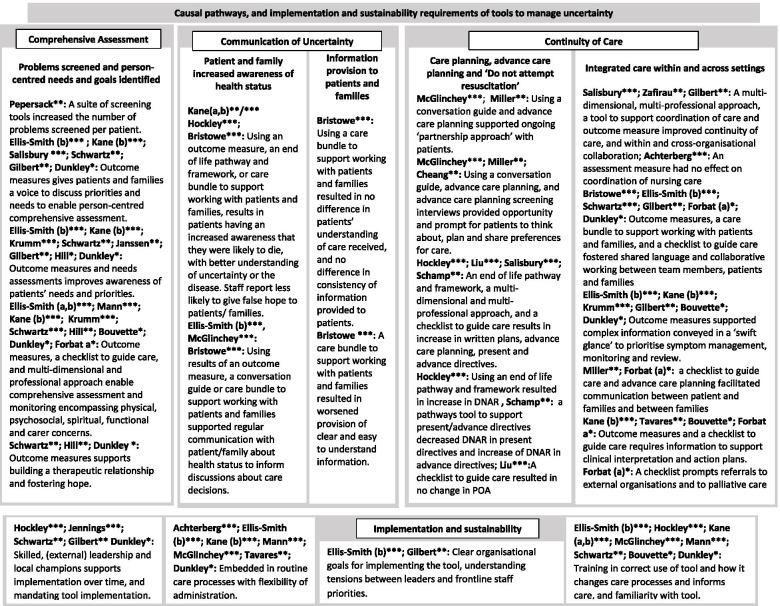
Causal pathways and implementation and sustainability requirements of tools to manage clinical uncertainty. Legend: Quality rating *** High quality; ** Medium quality; * Low quality

The causal pathways formed three overarching areas informed by our conceptual framework comprising: comprehensive assessment of the patient as a person and their family; communication with the patient and their family and continuity of care (Fig. 2).

#### Comprehensive assessment of the patient as a person and their family, and enhanced understanding of patients’ priorities and needs

Our findings suggest that using tools improved practitioners’ awareness of patients’ priorities and needs through facilitating and enabling a systematic and structured discussion. Most studies used validated outcome measures to support comprehensive assessment, encompassing multiple domains of health in a single multidimensional tool such as POS, or a battery of standardised assessment tools, such as CGA. Comprehensive assessment sought to move beyond the biomedical model to encompass unstable or unmet symptoms, psychosocial and spiritual concerns, and to identify the patient’s priorities and needs [19, 24–26, 30, 32, 35, 36, 50, 55, 60, 62]:

*‘It’s just so different from what you actually think and it’s quite frightening actually. You opened your eyes as to how complicated the human being is, totally and utterly. And [laughing] we don’t know it all and we never will. And people are just … [they] just live such different lives, their whole experience of life is so different from others.’ Nurse* [35] *(MVQOLI).*

The use of tools increased attention on the importance of person-centred care and legitimatised spending time with the patient to understand what mattered to them [19]. The time spent as a result of using the tool may support the development of a therapeutic relationship and enhance discussions that may be challenging for practitioners or the patient [35, 36]. These mechanisms are linked to perceptions of improved symptom management and psychosocial outcomes, for example patient empowerment [19, 22, 50]. Using a tool gave patients a voice to communicate, in a systematic way, with practitioners to support assessment and enabled patients to be more actively involved in the clinical consultation [21, 26, 35]:

*‘You never think of what’s wrong with you and how you’re feeling about it or has it improved, has it got worse, and should you do something different. I would think this [IPOS] is very good ‘cause, as I said, it makes you pinpoint exactly how you’re feeling … and what you can do or what you can’t do to improve it.’* [21] *(Patient 10, female NYHA III, HFmrEF).*

This facilitated consideration of areas practitioners and/or patients may not have otherwise discussed [22, 26, 35, 50, 55] and challenged practitioners’ perception of patients’ problems, shifting care and treatment to priorities for the patient [19, 22, 36].

Quantitative data supported these qualitative findings, indicating improved discussion of concerns important to patients [25] and improved screening of problems [42]. A high quality study tested the effectiveness of the 3D approach, an intervention targeting all domains [25]. In this Randomised Controlled Trial (RCT), 42% of patients in the intervention group reported that they almost always discussed the problems most important to them, compared to 26% in the control group, adjusted odds ratio (1.85 (95% CI 1.44–2.38), *n* = 1211). One medium quality study examined the use of a suite of tools, the Minimum Geriatric Screening Tools, to support comprehensive assessment [42]. The study demonstrated improvement in screening of problems after implementation of the intervention compared to before, with a mean increase of 3.2 (SD 1.8, *p* < 0.0001) problems per patient screened (*n* = 326).

#### Communication of clinical uncertainty with patients and families

Tools supported communication with patients and their families about the illness, changes in clinical presentation, progression of the disease and prognosis and empowered patients to engage in their own care. However, there was evidence that communication is challenging to do well and risks negative patient and carer experience. Tools targeting communication included outcome measures to facilitate discussion [21, 22, 24], end of life pathways and frameworks [53], a conversation guide [60], and a care bundle, called the Amber Care Bundle focusing on improving care and outcomes for hospitalised patients nearing the end of life and their families [47].

Tools improved communication with patients and families, supported improved understanding of the disease, and resulted in patients taking a more active role in understanding their disease [22, 60] and understanding of uncertainty [47]. Tools appeared to enhance communication with families by making routine the requirement for practitioners to update them on what to expect [47].

*‘one of the doctors actually rung me from home at nine o clock at night once because she realised she’d forgotten or hadn’t had a chance to come and see me so that was … was really nice and that was much appreciated.’ daughter of a man with lung cancer* [47] *(AMBER care bundle).*

Quantitative data provided mixed support for the qualitative findings. A high-quality study examined the experiences of receiving the AMBER care bundle [47]. As a result of the intervention, patients in the intervention group were more aware of nearing the end of life (72% intervention vs. 48% control, *p* = 0.038) (*n* = 80) and recalled discussion with practitioners about dying (59% intervention vs. 32% control, *p* = 0.043) (*n* = 63). However, families in the intervention group found information provided about the patients’ conditions less clear and easy to understand (51% intervention vs. 69% control, *p* = 0.044) (*n* = 80). There was no effect of the intervention on improving understanding of the care received (38% intervention vs. 50% control, *p* = 0.463) (*n* = 89) or consistency of information (45% intervention vs. 52% control, *p* = 0.253) (*n* = 90).

#### Continuity of care

Tools targeting this domain comprised a mix of outcome measures [19, 20, 22, 24, 33, 50, 55, 62], frameworks, pathways, and checklists [29, 30, 53, 58]; and those to facilitate discussions [60], support transfer of care across settings [59], support care planning and advance care planning [43, 45, 58], and support a multi-dimensional, multi-professional approach [25]. The use of tools was considered to support care planning and advance care planning; and to enhance clinical decision making and communication within and across clinical teams, and between clinical teams and patients and families. Use of a tool supported the summarising of the complexity of comprehensive assessment to an accessible format [19, 22, 24, 50, 55, 62] to enable rapid assessment, monitoring and review over time. This occurred by supporting processes of systematic collection of information, and planning of care and treatment [24, 30, 55, 62]:

*‘I used it [PSAR] on one of my patients who’d been having long-term pain … I liked it for her because it could monitor all of her other symptoms. She had a lot of other symptoms that went along with her pain as well’ Nurse in community agency* [62] *(PSAR).*

Tools supported collaboration with patients and families, including care planning and advance care planning [35, 43, 45, 60]; facilitating a ‘partnership approach’ [45, 60]. Patients appreciated the opportunity to consider, make and share decisions about their future care [43, 45]:

*‘I’m really pleased you came … It’s important to think about this* [ACP] *at my age … I hadn’t really thought about it before. … I want to speak with my niece about it. I want to think about whether I should be revived if my condition is really poor. Can you come back again?’ Patient* [43] *(ACP).*

A tool to support a multi-disciplinary approach sought to enable the contribution by all practitioners [30, 47], recognise their contribution [19, 30], and provide a common language for integrated working within and between care providers [24, 30], and between families [30]:

*‘We can meet with the families and we can get that plan in place and I think it’s really, really important, really decreases the amount of time people spend in hospital. For the elderly, it’s very traumatic to be taken to hospital when you’re unwell […] and we can manage it here, manage their pain, do the symptom management’ Manager, site 1* [30] *(Palliative Care Needs Rounds).*

Display and interpretation of item scores for each patient, with a benchmark for what constituted unstable symptoms or concerns and requirement for intervention facilitated changes to care provision [20, 22]. To impact on care, tools were required to include measurable and actionable items with clear clinical interpretation, for example, IPOS used in chronic heart failure indicated the need to address all individual items with scores ≥3, and to use clinical discretion for individual scores ≤2 [22]. The method of displaying the information gathered was crucial to maximise the usefulness of the observations, or reports gathered to inform clinical priorities and review [55, 62]. For example, a visual graphical map of pain and symptom intensity and summary enabled review by practitioners and involvement of patients (PSAR [62]). Conversely, completing a form alone would not inform or change clinical practice [24]:

*‘You know if we are because we’re in the situation where we’re thinking everybody’s exactly the same and then suddenly the data comes back saying actually you aren’t identifying that there have been quite significant changes which are written down but nobody’s doing anything about. Because the problem with care plans is you write things down but you don’t necessarily act on them’ Manager B3001.1* [24] *(IPOS-Dem).*

Quantitative results supported the qualitative findings. A high-quality study testing the effectiveness of the 3D approach in an RCT, showed a difference between the intervention (23%) and control (15%) group of patients reporting having a written care, health or treatment plan, adjusted odds ratio 1.97, (*p* = 0.001) (*n* = 1246) [25]. While another high-quality study of implementation of the Gold Standards Framework and Liverpool Care Pathway in care homes found increase in ACP from before intervention (4%) to after intervention (53%) (*p* < 0.0001) and demonstrated an increase in Do not attempt cardiopulmonary resuscitation (DNACPR) orders from 15 to 72% (*p* < 0.0001) [38]. A high-quality step wedge RCT, on the effectiveness of the Palliative Care Needs Rounds (*N* = 1700), detected an increase in ACP documentation from 30% (*n* = 208) in the control phase to 42% (*n* = 263) in the intervention phase (*p* < 0.01) [29], but not in appointment of a Power of Attorney (control phase 78% *n* = 208 versus intervention phase 74% (*n* = 263, *p* = 0.20).

A medium quality study of the Pathways tool using a before and after design demonstrated increased completion of present directives from 76.8 to 99.3% (*p* < 0.0001) and increased completion of advance directives from 35.6 to 100% (*p* < 0.0001) and a decrease of DNACPR in present directives from 48 to 38% (*p* < 0.071), but significant increase in DNACPR from 26 to 66% (*p* < 0.0001) in advance directives [58]. There was evidence that tools could improve coordination of care within and across care settings [25, 33, 59]. A medium quality study [59] examining use of a Transfer form to improve care transitions (Pre-intervention *N* = 130, Post-intervention *N* = 117) found the tool decreased discordance of advance directives between long term care facilities and hospital before (26.7%) and after (16.3%) the tool was implemented (*p* = 0.038). The Transfer form also decreased discordance between Emergency Department (ED) and hospital floor, before (26.7%) and after (16.3%) (95% CI, 0.050–0.299), and between hospital floor and long-term care facility (LTCF), before (40.0%) and after (27.1%) (95% CI, 0.143–0.437), but not between LTCF and ED, before (6.7%) and after (2.7%) (95% CI, − 0.007-0.103) [59]. A high-quality study testing the effectiveness of the 3D approach, an intervention targeting all domains, found more patients in the intervention group (42%) compared to the control (29%) reported that support and care is almost always joined up, adjusted odds ratio 1.48 (*p* = 0.0006) (*n* = 1217) [25]. Patients in the intervention group showed improved continuity of care in one measure versus the control group, adjusted difference 0.08 (95% CI, 0.02–0.13) (*n* = 1489), but not across all measures of continuity, adjusted difference, − 8.76 (95% CI, − 18.07-0.55) (*n* = 1489) [25]. However, a high-quality study demonstrated that using the RAI (*N* = 348) to increase coordination of care in nursing homes, did not detect an effect on overall coordination of care between the study arms (mean difference 2.8, 95% CI, − 0.28 – 5.82) [33].

### Evidence of effectiveness

**Fig. 3:**
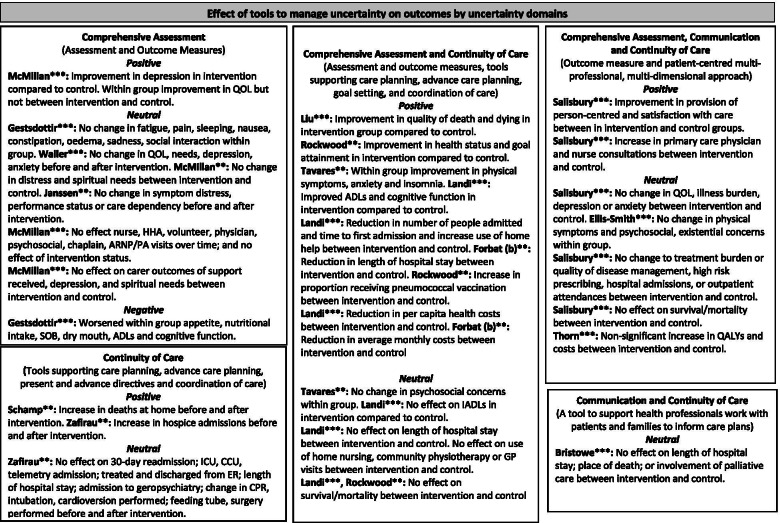
Effect of tools to manage clinical uncertainty on outcomes by domains. Legend: ARNP – advanced registered nurse practitioner, ADLs – activities of daily living, CCU – critical care unit, CPR – cardiopulmonary resuscitation, GP – general practitioner, HHA – home health aide, IADLs – instrumental activities of daily living, ICU – intensive care unit, PA – physician assistant, SOB – shortness of breath, QOL – quality of life. Quality rating: ***High quality; **Medium quality; *Low quality

Eleven studies tested the tools’ effect on the stated outcomes (Table 2, Fig. 3). We grouped and analysed these by the clinical uncertainty domain(s) we were seeking to address.

#### Tools targeting all domains

**Fig. 4:**
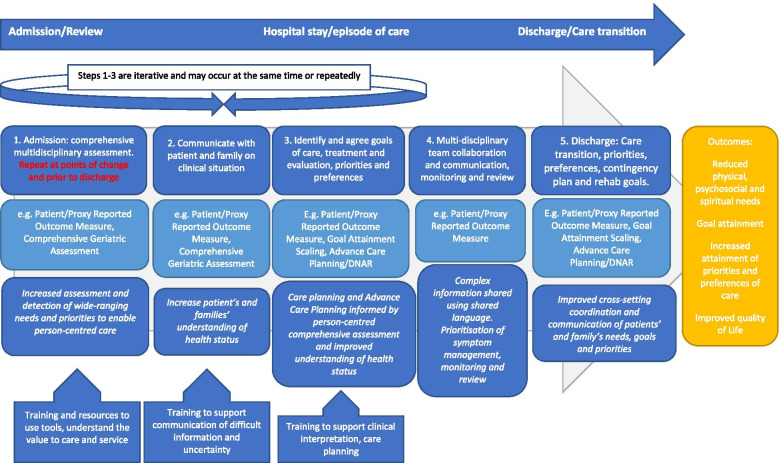
Logic model demonstrating how tools may support management of clinical uncertainty in older people

Three high quality studies examined the effectiveness of two tools targeting all three domains, the 3D approach [25, 27] and the IPOS-Dem [24]. The results indicated effectiveness at improving person-centred care and increasing consultations, but no effect at improving quality of life, symptom burden, treatment burden, or hospital use, or cost-effectiveness. The effectiveness [25] and cost-effectiveness [27] of the 3D approach was examined in a high-quality pragmatic cluster RCT (*N* = 1546). The 3D approach was found effective between the intervention and control group in multiple measures of person-centred care and patient satisfaction and in increasing nurse and primary care physician consultations [25]. But it did not have an effect on quality of life, illness burden, depression, anxiety, hospital admissions, high risk prescribing, hospital outpatient attendances or treatment burden. The 3D approach was not cost-effective with small increases in Quality Adjusted Life Years and costs (Table 2, Fig. 4). The IPOS-Dem was evaluated in a high- quality mixed methods process evaluation (*N* = 32). It was not powered to detect effectiveness and found no effect at reducing symptom burden (Table 2, Fig. 3) [24].

#### Tools targeting comprehensive assessment

Three high quality [31, 34, 49] and two medium quality [32, 35] studies examined the effectiveness of tools targeting comprehensive assessment. The interventions included: a package of tools with feedback of results to the care team [49], the MVQOLI [35]; the Needs Assessment Tool: Progressive Disease - Cancer (NAT:PD-C) [31]; the Needs Assessment Tool: Progressive Disease – Heart Failure (NAT:PD-HF) [32] and the Inter Residents Assessment Instrument-Palliative Care (InterRAI-PC) [34]. Tools targeting comprehensive assessment demonstrated low strength of evidence of improved quality of life and improving mood, but no effect at improving all other outcomes, with some outcomes worsening over time in deteriorating populations.

A package of measures with feedback of results to the care team was evaluated in a high quality RCT [49] and the MVQOLI was evaluated in a medium quality before and after quasi-experimental study [35]. Both tools demonstrated no effect at improving quality of life between the intervention and control group, but there was some within-group improvement in quality of life in the intervention group [35, 49]. The intervention improved depression but showed no effect at improving distress, health service use, or carer outcomes [49].

Two versions of the Needs Assessment Tool: Progressive Disease (NAT:PD) were evaluated in two studies. The NAT-PD-C was evaluated in a high quality interrupted time series study (*N* = 114), but no effect was found in changes to quality of life, depression, anxiety, or needs [31]. The NAT:PD-HF (*N* = 17) was evaluated in a medium quality pre-test/post-test pilot study and demonstrated no effect at reducing symptom distress, dependency, or carer outcomes, while participants’ health status worsened [32]. The InterRAI-PC was evaluated in a high quality prospective longitudinal study (*N* = 81) [34]. There was no change in symptom control and participants showed worsening physical and cognitive function over the duration of the study (Table 2, Fig. 3).

#### Tools targeting comprehensive assessment and continuity of care

Five publications, reporting four studies [20, 28, 29, 37, 56], evaluated tools targeting comprehensive assessment and communication. Tools targeting these two domains demonstrated effectiveness at improving quality of death and dying, clinician’s global assessment, goal attainment and symptom burden. There were mixed effects on function and health service use, but demonstrated cost saving.

The Palliative Care Needs Rounds Checklist was evaluated in high/medium quality step-wedged RCT reported in two publications [28, 29] (*N* = 1700) and showed effectiveness on quality of death and dying [29], and reducing length of hospital stay and saving costs [28]. The Minimum Data Set for Home Care (MDS-HC) was evaluated in a high quality RCT (*N* = 176). It demonstrated effectiveness between groups on the outcomes of Activities of Daily Living (ADL), cognitive function, hospital admissions, and use of community home health, and cost saving, but not on instrumental activities of daily living, hospital stay, and use of other community services [56]. A medium quality RCT evaluated a combined intervention of the CGA and Goal Attainment Scale (GAS) (*N* = 182) [37] that showed effectiveness on clinician’s global assessment, goal attainment, and pneumococcal inoculation, but not on hospital admissions. A medium quality observational study, piloted (*N* = 57) and evaluated (*N* = 317) the POS [20]. Following pilot and modification, it was evaluated in routine care with participants demonstrating a reduction of symptoms between timepoints.

#### Tools targeting continuity of care

Two studies evaluated two tools targeting the domain of continuity of care [58, 59]. These studies demonstrated effectiveness at increasing hospice admissions and increasing deaths at home, but no effect on other health service outcomes. The Pathways tool was evaluated in a medium quality pre-test (*n* = 33)/post-test (*n* = 49) and was effective at increasing deaths at home [58]. The Resident Change in Condition Assessment/Transfer form was evaluated in medium quality pre-test (*n* = 130)/post-test (*n* = 117) study. It showed some effect on hospice admissions but no effect on any other of the health service outcomes including hospital admission and length of stay [59].

#### Tools targeting communication and continuity of care

One high quality comparative observational study found no effect on hospital length of stay, place of death and palliative care involvement [47].

### Implementation and sustainability

We found three overarching themes related to the implementation and sustainability of tools; leadership, cost and workforce implications and embedding of tools into care processes.

#### Skilled leadership to enable innovation in clinical practice

Skilled leadership and clear organisational goals across stakeholders [24, 33, 50] was essential in the uptake and ongoing use of tools, specifically when a ‘cascade’ style was adopted. This meant that leaders or champions used the tools first and then supported use with others [50, 53]. The influence of leadership was not always limited to internal organisations [50, 53]. External leadership, including at provincial or regional level, appeared influential and was often accompanied by more support [50, 53, 54]. These dedicated external teams were comprised of health care practitioners or administrative staff with a remit to plan and test changes in care processes, sometimes using specific quality improvement models to support the innovation [19, 50, 53]. Participating in an initiative beyond the service with opportunities to share experiences was highly valued and perceived as supporting use [50]:

*‘It was reassuring to discover that others around the province were experiencing the same issues’ Team member* [50] *(Palliative Performance Scale and ACP).*

Senior organisational leaders incorporated an infrastructure to support use across services and regions [36, 50]. However, standardising processes across several organisations during implementation is not straightforward and was described as *‘building a plane in flight’* [50].

#### Cost and workforce implications

We found three areas of cost and workforce implications: training, potential benefit to practitioners and organisations, and barriers due to practitioners’ time and resources and infrastructure.

Training was considered an important component of implementation. It included how to use the tool [21, 22, 24, 26, 33, 36, 53, 57, 62], understanding how the tool may support care processes [26, 36, 53], and exposure to the tool prior to implementation [36, 53, 62]. Training ranged from provision of a manual [24], ‘high facilitation’ with frequent site visits and significant levels of in-house training [30, 53], or locally provided training at the discretion of site lead. A single training session was sometimes perceived as insufficient, with practitioners requesting follow up training to ensure they were ‘doing it right’ [26].

Some studies reported the benefits of tools to support training and education in assessment [19, 24, 30], the care that they provide [30, 53], or the opportunity to reflect on their own beliefs about their patients’ circumstances [35]. One example was the Palliative Care Needs Rounds intervention incorporating education of practitioners at each Needs Rounds. This included identifying palliative care needs and discussing palliative care with families and improving knowledge, skill and confidence [30]:

*‘[The model of using Needs Rounds is] about how many [staff in residential care] can do a great job. Spreading it so that the knowledge and skill spread.’ Specialist palliative care clinician* [30] *(Palliative Care Needs Rounds).*

The effectiveness of the Palliative Care Needs Rounds on improving practitioners’ capability was tested (*N* = 1700 care home residents) [29]. Practitioners’ self-reported capability was examined using the Capacity to Adopt a Palliative Care Approach (CAPA) tool. CAPA scores were compared before the intervention began and with scores 6 months later. There was an improvement in CAPA scores from 29.4 pre-intervention (*n* = 84 practitioners) to 34.2 post-intervention (*n* = 161 practitioners), with a difference of 4.7 (95% CI: 2.7–6.7) [29].

Introducing tools created wider benefit than for patients and training of practitioners. Use of tools also contributed to practitioners feeling empowered and valued [19]. Tools were seen to benefit the service when used as an audit tool or outcome measure [19, 24] and when there were sufficient resources to analyse service level data [19, 33, 50]. Used in this way, services were able to demonstrate the care they were providing for quality inspections or funding purposes [19, 24]:

*‘and I know it’s more work, but even if it’s only a little bit, it’s still more work regardless of a little or a lot but I think things like this which, I don’t mean this selfishly, doesn’t just look after the clients, it promotes us, it promotes the care we’re giving, it promotes the way in which we work, so you know, I don’t think it shouldn’t be done. I think it’s something that all homes should do’ Manager C1005* [24] *(IPOS-Dem).*:

Introducing tools had resource implications, particularly on practitioners’ time. Most tools within this review required completion or practitioners’ assistance, placing additional burden on already stretched practitioners [19, 24, 26, 35, 38, 50], with challenges when there were staff shortages [19, 26]. Factors to facilitate uptake were senior acknowledgement of the extra time required to implement the tools, the whole team being engaged in the implementation and ensuring that the data is relevant and informs care [19, 32, 50] and targets the patients who would benefit most [26]. There was some evidence that practitioners became more skilled, efficient and faster using tools over time [19, 24, 36].

Identifying uncertain prognosis and therefore who might be at risk of dying and benefit from the intervention proved challenging, resulting in patients who had more certainty of dying being recruited [48]. Practitioners reported concerns about initiating palliative care discussions with patients, including taking away hope [32]. Similarly, some patients and families demonstrated unwillingness to discuss palliative care or did not view the condition as life limiting [32]:

*‘It’s so difficult sometimes. For example when nobody has discussed the end-of-life before. And then I have to introduce such a questionnaire’ Heart failure nurse specialist* [32] *(NAT:PD-HF)*

Challenges resulted from tools assessing symptoms beyond practitioners’ knowledge, skills, or of little relevance to the population [22, 23, 26, 32, 62]. Practitioners identified the importance of communication training [26], understanding how to identify when patients could benefit from palliative care [55] and concerns about meeting identified needs within available resources and level of competencies [21, 26, 32, 35, 36, 50]:

*‘but it would be a difficult one to broach, I suppose it would open it up for you, you could start the conversation. … you could maybe guide them towards their priest, or maybe something like that. But I think I’d only be able to discuss that with them … it would be a difficult one’ Nurse 02* [21] *(IPOS)*

#### Embedded into care processes e.g. part of routine care

The extent to which the measures were embedded into routine care was closely linked to the level of involvement and support provided by external and internal organisational leaders. Tools were administered at different points within the care process such as at times of care planning or review [24, 26, 33], at routine clinic appointments [21, 22] or through daily electronic monitoring while at home [52] or in a care home [55]. Challenges to embedding tools into routine practice resulted in reduced uptake and unnecessary duplication [26]. Used too frequently, tools also became burdensome [19, 26] and affected how useful the tool was to clinical care. Flexibility was preferred in how frequently tools were used according to patient need, with increased use when there has been a change in health status [19, 20, 24, 26, 33, 35]:

*‘For some people it might vary, some people you might need to do it every day (Care home staff C1007) . . . […]. . .whereas some people you might do it once a month, while some you have to do it weekly’ Care home staff* [24] *(IPOS-Dem)*

There was reluctance to change from tools with which practitioners were familiar [50]. Whole team involvement in using tools within existing structures and processes facilitated integration [19, 24, 26]. While tools sought to improve continuity of care and collaboration, disjointed team working contributed to barriers to using tools [26, 32]:

*‘We discuss with the patient, before the doctor comes, that they should realise that maybe this is it, and it won’t get better … Yes, we try to introduce this and then the cardiologist comes in and says: we will do this and that and here is a prescription and then I think: what is this?’ Heart failure nurse specialist* [32] *(NAT:PD-HF)*

### Tool properties supporting implementation and sustainability

Three tool properties were identified that supported use in clinical care: tools that supported and promoted person-centred care and provided value to care, ease of use and feasibility and psychometric properties.

#### Promoting person-centred care and adding value to care

Patients valued tools that provided the opportunity to discuss important issues and identify areas that needed attention [35] and those that provided the opportunity to discuss and share wishes and facilitated thinking about wishes not previously considered. Patients recognised the value of tools to ensure their wishes were carried out and support their families [43, 45]:

*‘they seem to be relieved that they’ll know what my wishes are’ Patient 10* [43] *(ACP)*

Tools were valued and more acceptable when practitioners could see how they supported person-centred care and improved care processes [19, 26, 30, 36, 44, 60] and when they facilitated a dialogue and conversation with patients and family members:

*‘Sometimes communication is the last thing that you think about and it should be the first thing you think about, because you find out so much more’ Healthcare Assistant 04, Hospice* [19] *(POS)*

Not all tools were seen to support person-centred care, and needed to be used in ways that enhanced assessment without replacing clinical judgement [26, 52]:

*‘I think there has always got to be scope for looking at that particular patient and looking at their own specific needs in maybe a slightly different...much more holistic way than that tool allows.’ Clinician 12* [52] *(ESAS)*

Practitioners reported challenges when patients wanted them to make decisions [26] and struggled to engage with discussions about future care [32] and, while tools were frequently seen to facilitate communication, this did not mean that the quality or sensitivity of communication was improved [47]:

*‘The doctor told me we are in a situation of diminishing returns and ought to let nature take its course … this was so blunt … I couldn’t sleep for two days’ , man with lung cancer* [47] (AMBER care bundle)

#### Ease of use and feasibility

Tools were more acceptable to practitioners when they could be used by any member of the team and supported the whole team working together [19, 20, 47]. It was important that tools were brief, concise, easy to use and flexible to administer [19, 26, 32, 35, 38, 41, 44, 45, 55, 60–62]. This included using lay, clear, simple and informal language [23, 41, 46, 60, 61] and training in accessible formats such as short videos [23]. Layout was also considered important for ease of use [61, 62].

#### ‘A trusted measure’ – psychometric properties

Study participants did not generally use psychometric terms but considered psychometric properties important in the tools that they were using. Tools need to be rigorously developed to ensure that they were valid and relevant to the population and setting [19, 20, 23, 38, 46, 62]:

*‘What about psychological pain? Depression? Spiritual pain? Social pain? I think all these other aspects of pain are definitely going to impact the physiological pain and pain control, so if we don’t look at those, then I think we’re missing the boat on rating pain, physical pain.’ Community agency* [62] *(PSAR)*

There was some evidence of the importance of building on science and using established, known and trusted and validated measures [23, 24, 33, 55]. Participants identified items that were less useful [62], or tools that were challenging in terms of reliability [20, 40]. Challenges resulted from tools used late in the disease trajectory or when patients were very ill or cognitively impaired, resulting in less confident assessment [23, 24, 36, 55, 61]. Reliable assessment was considered important for the tool to be trusted to inform clinical care [24]:

*‘and [care home staff have] got the time to do it honestly, truthfully, then yes because anyone that needs to look at this whether it be GP, ambulance, consultant, relative, they know exactly what is going on’ Family B3006* [24] *(IPOS-Dem)*

## Discussion

### Summary of findings

Our findings intend to advance the conceptual understanding of clinical uncertainty to a greater understanding of how it is managed for older people towards the end of life. Our logic model (Fig. 4) demonstrates the causal mechanisms and linkages to improve outcomes, how tools are used to enhance care processes across an episode of care and requirements to use in clinical care.

Our findings show that tools that target comprehensive assessment and continuity of care improve outcomes of quality of death and dying, clinician’s global assessment, goal attainment and symptom burden. However effect across the studies was variable on outcomes of psychosocial concerns, functioning, and service use and costs. These tools facilitate a comprehensive assessment of a person’s priorities, needs and wishes and then inform and support decisions about care, inform advance care plans and support joint working between families and care teams and across teams and organisations. Our findings indicate limited evidence of benefit of tools that target comprehensive assessment alone. In this domain, only the McMillan et al. study (2011) demonstrated effectiveness on the outcome of depression [49]. However, even studies that evaluated tools targeting all three domains [24, 25, 27] showed limited effectiveness with only one study reporting effect on care provision [25]. The domain of communication was the least targeted and therefore least evaluated. It also proved to be the domain most challenging to address. Our findings suggest that practitioners may require training on how to act upon their assessment, including interpreting scores and developing and initiating action plans, and training in communication of clinical uncertainty. Training may also be needed to support implementation, including how to use tools, how tools may work to support care, and how to embed tools into routine care.

### Communication of clinical uncertainty

We found this as the most challenging area to do well, and the area where the fewest tools have been developed. This is perhaps unsurprising. Clinical uncertainty can be challenging for practitioners to communicate and manage within teams, between teams, and between teams and families, and may result in conflict [5, 13]. Tools are not able to replace good quality skilled communication. This finding is supported by other studies in uncertainty, where excellent communication skills were found to be required, particularly at time of conflict such as uncertain prognosis [5, 67]. Poor communication may, at best, negate any positive effect of the tool, and may cause worse outcomes and distress for patients and families. However, some tools have been developed to ensure that practical steps can be taken to aid communication, and can result in better outcomes for patients and families in the intensive care setting [7] and for patients with incurable cancer [68]. Other important training interventions have been developed including, for example, VitalTalk, to support communication with those living with serious illness [69]. Patients and families can only truly be involved in the care decisions when they are fully informed and understand all options, and it is therefore essential that practitioners have training to support these potentially difficult conversations.

### Delivery and review of care plans

Tools can support care planning by providing a structured process of assessment, and structured process of identifying patient goals and priorities. They may also have an important role in monitoring and reviewing care plans, by comparing scores over time, and support integrated working within and across teams. We found that this structured process can lead to improved outcomes. However, tools alone may not always be sufficient to change the way that care is delivered, and there is evidence that care plans may not always translate into changes in daily care [70, 71]. We found that there is a requirement for tools to provide clinically relevant information and prompt clear action plans. It is also essential that practitioners have the clinical skills and resources to action care plans, and that tools are embedded into care processes to support and facilitate delivery of care.

### How our findings compare to comprehensive geriatric assessment (CGA)

Our review findings and logic model overlap and incorporates processes of CGA [72, 73], and indeed many articles identified intended to comprise a CGA intervention. We found that the use of tools may include and facilitate some of the intended CGA processes, including that of structured comprehensive assessment, care planning and working towards patient goals [72, 74]. However, our conceptual underpinning of clinical uncertainty meant that we included other tools, specifically tools to communicate clinical uncertainty to patients and families, and tools to support communication within and across teams and services. Another important difference is that the majority of CGA interventions involve multi-disciplinary service delivery models, rather than the use of tools, meaning that many did not meet the inclusion criteria. Using tools may support CGA processes and be more feasible to implement, particularly in the non-acute hospital sector, but risk losing the specialist multi-disciplinary expertise that a service delivery approach brings.

### Strengths and limitations

Our review has several strengths and limitations. An important strength is the initial development of a conceptual underpinning of clinical uncertainty. This informed our methods, data analysis and interpretation. The use of an extensive mixed methods review, using robust underpinning methodology, enabled development of a logic model to advance conceptual understanding and application for clinical practice [17, 66]. However, there are limitations. First, decisions about whether interventions were primarily a tool and decisions about whether the intervention targeted clinical uncertainty were unavoidably subjective. As such, all those with uncertain inclusion eligibility were discussed within the project team. Due to the nature of the review, we included multiple study designs and used quality assessment, rather than risk of bias assessment. This meant that strength of evidence and risk of bias was variable when reporting effectiveness. However, we have reported the study designs to assist the reader in interpreting the results of effectiveness. Finally, the quality of the study does not reflect the generalisability of the study findings. In particular, the majority of the included studies were conducted in high income countries and may not be generalisable to low and middle-income countries (LMICs).

### Clinical and research implications

We have identified how tools can change care processes to improve outcomes. We have also identified the properties tools need to be implemented and sustained in clinical practice. These include tools that are person-centred, target multiple domains and provide an actionable treatment plan. Tools that are brief and easy to use and developed for the target population and are used nationally or internationally with strong psychometric properties were also identified as easier to implement and sustain. There are multiple internationally established tools such as the RAI [33, 34, 75, 76] and the POS [77–79]. It is important that the science builds upon established tools and the existing evidence, and that future areas of research link to the logic model. Key areas of research need to include high quality RCTs, using the logic model to inform key processes, causal mechanisms and intended outcomes and implementation requirements. Further development work to understand the causal mechanisms and linkages to outcomes in wider contexts including LMICs is also indicated. Intervention development work needs to be done to support practitioners communicating clinical uncertainty to patients and families, including a training component.

## Conclusion

This review moves our conceptual understanding of uncertainty into its applied management in the clinical care of older people towards the end of life. We have developed a logic model to demonstrate the key causal pathways of how tools to manage clinical uncertainty may work and linkages with the intended outcomes. Person-centred tools are essential to improve care and should be implemented into routine practice. Communication of clinical uncertainty is the most challenging and most neglected area. Wider consideration is required of how best to enable informed patient and family involvement in decisions about care and treatment.

## Supplementary Information

**Additional file 1.** Conceptual framework.

**Additional file 2.** PRISMA checklist.

**Additional file 3.** Full search strategy.

## Data Availability

All data generated or analysed during this study are included in this published article [and its supplementary information files].

## Abbreviations

ACP: Advance Care Plan (or planning)
ADL: Activities of daily living
AKPS: Australia-modified Karnofsky Performance Status
CAPA: Capacity to Adopt a Palliative Care Approach
CARE: Consultant and relational empathy
CAM: Confusion Assessment Method
CCI: Charlson Comorbidity Index
CCU: Critical care unit
CDS: Care dependency scale
CES-D: Center for Epidemiological Study-Depression Scale
CGA: Comprehensive Geriatric Assessment
CHF: Chronic heart failure
CPR: Cardiopulmonary resuscitation
CSDD: Cornell Scale for Depression in Dementia
DNACPR: Do not attempt cardiopulmonary resuscitation
DNAR: Do not attempt resuscitation
ED: Emergency department
EDIZ: Experienced Burden of Informal Care
EORT QLQ-C30: European Organization for Research and Treatment of Cancer Quality of Life Questionnaire
EQ-5D (− 5 L): EuroQol-5D (5 level)
ER: Emergency room
ESAS: Edmonton Symptom Assessment Symptom
FACQ-PC: Family Appraisal of Caregiving Questionnaire Palliative Care
FRAIL screen: Fatigue, Resistance, Ambulation, Illness and Loss of weight screen
GA: Geriatric Assessment
GAS: Goal Attainment Scale
GP: General Practitioner
GDS: Geriatric Depression Scale
GOC: Goals Of Care
GPN: General practitioner nurse
HADS: Hospital anxiety and depression scale
HQLI: Hospice Quality of Life Index
IADL: Instrumental activities of daily living
ICER: Incremental Cost-Effectiveness Ratio
ICU: Intensive care unit
IQCOD-SF: Informant Questionnaire Cognitive Decline – Short Form
InterRAI-PC: Inter Resident Assessment Instrument – Palliative Care
IPOS: Integrated Palliative care Outcome Scale
IPOS-Dem: Integrated Palliative care Outcome Scale for Dementia
IQR: Interquartile range
ISAR: Identification of Seniors at Risk
(Lawton) IADL: (Lawton) Instrumental Activities of Daily Living
LCP: Liverpool Care Pathway
LTCF: Long term care facility
LMIC: Low and middle income country
MDS: Minimum Data Set
MDS-HC: Minimum Data Set for Home Care
MGST: Minimum Geriatric Screening tool
MIDOS: Minimal Documentation system for Palliative Care
MLHFQ: Minnesota living with heart failure questionnaire
MMSE: Mini Mental State Examination
MNA-SF: Mini Nutritional assessment – short form
MSAS: Memorial Symptom Assessment Scale-Revised
MVQOLI: Missoula-VITAS Quality of Life Index
NAT:PD-C: Needs Assessment Tool: Progressive Disease – Cancer
NAT:PD-HF: Needs Assessment Tool: Progressive Disease – Heart Failure
NHS: National Health Service
NPI-q: Neuropsychiatric Inventory Questionnaire
PACIC: Patient Assessment of Care for Chronic Conditions
POLST: Physician Orders for Life-Sustaining Treatment
POS (−S): Palliative care Outcome Scale (−symptoms)
PRISMA: Preferred Reporting Items for Systematic Reviews and Meta-Analyses
PROM: Patient reported outcome measure
PSAR: Pain and Symptom Assessment Record
PSS: Personal social services
QALY: Quality-adjusted life year
QODD: Quality of death and dying
QOL: Quality of Life
RAI: Residents Assessment Instrument
RCS: Rapid Cognitive Screen
RCT: Randomised Controlled Trial
SD: Standard deviation
SF-36: Short Form survey
SNAQ: Short Nutritional Assessment Questionnaire
SSI: Social Support Instrument
TMT: Trail Making Test
VAS: Visual Analogue Scale

## References

[1] Fried LP, Tangen CM, Walston J, Newman AB, Hirsch C, Gottdiener J (2001). Frailty in older adults: evidence for a phenotype. J Gerontol A Biol Sci Med Sci.

[2] Gill TM, Gahbauer EA, Han L, Allore HG (2010). Trajectories of disability in the last year of life. N Engl J Med.

[3] Clegg A, Young J, Iliffe S, Rikkert MO, Rockwood K (2013). Frailty in elderly people. Lancet..

[4] Joint Health Surveys Unit, Social & Community Planning Research, Department of Epidemiology and Public Health, University College London. The health survey for England 2000: the general health of older people and their use of health services. London; 2002.

[5] Higginson IJ, Rumble C, Shipman C, Koffman J, Sleeman KE, Morgan M (2016). The value of uncertainty in critical illness? An ethnographic study of patterns and conflicts in care and decision-making trajectories. BMC Anesthesiol.

[6] Dalgaard KM, Thorsell G, Delmar C (2010). Identifying transitions in terminal illness trajectories: a critical factor in hospital-based palliative care. Int J Palliat Nurs.

[7] Higginson IJ, Koffman J, Hopkins P, Prentice W, Burman R, Leonard S, et al. Development and evaluation of the feasibility and effects on staff, patients, and families of a new tool, the psychosocial assessment and communication evaluation (PACE), to improve communication and palliative care in intensive care and during clinical uncertainty. BMC Med. 2013;11:213. 10.1186/1741-7015-11-213. 10.1186/1741-7015-11-213 PMC3850793 24083470

[8] Johnson Wright L, Afari N, Zautra A (2009). The illness uncertainty concept: a review. Curr Pain Headache Rep.

[9] Mishel MH (1981). The measurement of uncertainty in illness. Nurs Res.

[10] Mishel MH (1988). Uncertainty in illness. Image—J Nurs Scholarship..

[11] Mishel MH (1990). Reconceptualization of the uncertainty in illness theory. Image—J Nurs Scholarship.

[12] Etkind SN, Bristowe K, Bailey K, Selman LE, Murtagh FE (2017). How does uncertainty shape patient experience in advanced illness? A secondary analysis of qualitative data. Palliat Med.

[13] Goodman C, Froggatt K, Amador S, Mathie E, Mayrhofer A (2015). End of life care interventions for people with dementia in care homes: addressing uncertainty within a framework for service delivery and evaluation. BMC Palliat Care.

[14] Lin C-P, Evans CJ, Koffman J, Armes J, Murtagh FEM, Harding R (2019). The conceptual models and mechanisms of action that underpin advance care planning for cancer patients: a systematic review of randomised controlled trials. Palliat Med.

[15] Hong QN, Pluye P, Bujold M, Wassef M (2017). Convergent and sequential synthesis designs: implications for conducting and reporting systematic reviews of qualitative and quantitative evidence. Syst Rev.

[16] Moore G F, Audrey S, Barker M, Bond L, Bonell C, Hardeman W et al. Process evaluation of complex interventions: Medical Research Council guidance. BMJ. 2015;350:h1258. 10.1136/bmj.h1258. 10.1136/bmj.h1258 PMC4366184 25791983

[17] Popay J, Roberts H, Sowden A, Petticrew M, Arai L, Rodgers M (2006). Guidance on the conduct of narrative synthesis in systematic reviews. A product from the ESRC methods programme version.

[18] Evans C, Ison C, Ellis-Smith C, Nicholson C, Costa A, Oluyase A (2019). Service delivery models to maximize quality of life for older people at the end of life: a rapid review. Milbank Q.

[19] Dunckley M, Aspinal F, Addington-Hall JM, Hughes R, Higginson IJ (2005). A research study to identify facilitators and barriers to outcome measure implementation. Int J Palliat Nurs.

[20] Tavares AP, Paparelli C, Kishimoto CS, Cortizo SA, Ebina K, Braz MS (2017). Implementing a patient-centred outcome measure in daily routine in a specialist palliative care inpatient hospital unit: an observational study. Palliat Med.

[21] Kane PM, Ellis-Smith CI, Daveson BA, Ryan K, Mahon NG, McAdam B (2018). Understanding how a palliative-specific patient-reported outcome intervention works to facilitate patient-centred care in advanced heart failure: a qualitative study. Palliat Med.

[22] Kane PM, Daveson BA, Ryan K, Ellis-Smith CI, Mahon NG, McAdam B (2017). Feasibility and acceptability of a patient-reported outcome intervention in chronic heart failure. BMJ supportive &amp. Palliat Care..

[23] Ellis-Smith C, Evans CJ, Murtagh FE, Henson LA, Firth AM, Higginson IJ (2017). Development of a caregiver-reported measure to support systematic assessment of people with dementia in long-term care: the integrated palliative care outcome scale for dementia. Palliat Med.

[24] Ellis-Smith C, Higginson IJ, Daveson BA, Henson LA, Evans CJ (2018). How can a measure improve assessment and management of symptoms and concerns for people with dementia in care homes? A mixed-methods feasibility and process evaluation of IPOS-Dem. PLoS One.

[25] Salisbury C, Man M-S, Bower P, Guthrie B, Chaplin K, Gaunt DM (2018). Management of multimorbidity using a patient-centred care model: a pragmatic cluster-randomised trial of the 3D approach. Lancet.

[26] Mann C, Shaw ARG, Guthrie B, Wye L, Man M-S, Chaplin K (2019). Can implementation failure or intervention failure explain the result of the 3D multimorbidity trial in general practice: mixed-methods process evaluation. BMJ Open.

[27] Thorn J, Man M-S, Chaplin K, Bower P, Brookes S, Gaunt D (2020). Cost-effectiveness of a patient-centred approach to managing multimorbidity in primary care: a pragmatic cluster randomised controlled trial. BMJ Open.

[28] Forbat L, Liu W-M, Koerner J, Lam L, Samara J, Chapman M (2019). Reducing time in acute hospitals: a stepped-wedge randomised control trial of a specialist palliative care intervention in residential care homes. Palliat Med.

[29] Liu W-M, Koerner J, Lam L, Johnston N, Samara J, Chapman M (2020). Improved quality of death and dying in care homes: a palliative care stepped wedge randomized control trial in Australia. J Am Geriatr Soc.

[30] Forbat L, Chapman M, Lovell C, Liu W-M, Johnston N (2018). Improving specialist palliative care in residential care for older people: a checklist to guide practice. BMJ Support Palliat Care.

[31] Waller A, Girgis A, Johnson C, Lecathelinais C, Sibbritt D, Forstner D (2012). Improving outcomes for people with progressive cancer: interrupted time series trial of a needs assessment intervention. J Pain Symptom Manag.

[32] Janssen DJ, Boyne J, Currow DC, Schols JM, Johnson MJ, La Rocca HB (2019). Timely recognition of palliative care needs of patients with advanced chronic heart failure: a pilot study of a Dutch translation of the needs assessment tool: progressive disease - heart failure (NAT:PD-HF). Eur J Cardiovasc Nurs.

[33] Achterberg WP, Holtkamp CCM, Kerkstra A, Pot AM, Ooms ME, Ribbe MW (2001). Improvements in the quality of co-ordination of nursing care following implementation of the resident assessment instrument in Dutch nursing homes. J Adv Nurs.

[34] Gestsdottir B, Hjaltadottir I, Gudmannsdottir GD, Jonsson PV, Gunnarsdottir S, Sigurđardottir V (2015). Symptoms and functional status of palliative care patients in Iceland. Br J Nurs.

[35] Hill N (2002). Use of quality-of-life scores in care planning in a hospice setting: a comparative study. Int J Palliat Nurs.

[36] Schwartz CE, Merriman MP, Reed G, Byock I (2005). Evaluation of the Missoula-VITAS quality of life index-revised: research tool or clinical tool?. J Palliat Med.

[37] Rockwood K, Stadnyk K, Carver D, MacPherson KM, Beanlands HE, Powell C (2000). A clinimetric evaluation of specialized geriatric care for rural dwelling, frail older people. J Am Geriatr Soc.

[38] Parlevliet JL, Buurman BM, Pannekeet MM, Boeschoten EM, ten Brinke L, Hamaker ME (2012). Systematic comprehensive geriatric assessment in elderly patients on chronic dialysis: a cross-sectional comparative and feasibility study. BMC Nephrol.

[39] Basic D, Conforti D, Rowland J (2002). Standardised assessment of older patients by a nurse in an emergency department. Aust Health Rev.

[40] Mariano C, Williams G, Deal A, Alston S, Bryant AL, Jolly T (2015). Geriatric assessment of older adults with cancer during unplanned hospitalizations: an opportunity in disguise. Oncologist..

[41] Jadczak AD, Mahajan N, Visvanathan R (2017). The feasibility of standardised geriatric assessment tools and physical exercises in frail older adults. J Frailty Aging.

[42] Pepersack T, College of G, the Belgian Society for G, Geriatrics (2008). Minimum geriatric screening tools to detect common geriatric problems. J Nutr Health Aging.

[43] Cheang F, Finnegan T, Stewart C, Hession A, Clayton JM (2014). Single-centre cross-sectional analysis of advance care planning among elderly inpatients. Intern Med J.

[44] Silvester W, Parslow RA, Lewis VJ, Fullam RS, Sjanta R, Jackson L (2013). Development and evaluation of an aged care specific advance care plan. BMJ Support Palliat Care.

[45] Miller H, Tan J, Clayton JM, Meller A, Hermiz O, Zwar N (2019). Patient experiences of nurse-facilitated advance care planning in a general practice setting: a qualitative study. BMC Palliat Care..

[46] Sudore RL, Stewart AL, Knight SJ, McMahan RD, Feuz M, Miao Y (2013). Development and validation of a questionnaire to detect behavior change in multiple advance care planning behaviors. PLoS ONE [Electronic Resource].

[47] Bristowe K, Carey I, Hopper A, Shouls S, Prentice W, Caulkin R (2015). Patient and carer experiences of clinical uncertainty and deterioration, in the face of limited reversibility: a comparative observational study of the AMBER care bundle. Palliat Med.

[48] Koffman J, Yorganci E, Yi D, Gao W, Murtagh F, Pickles A (2019). Managing uncertain recovery for patients nearing the end of life in hospital: a mixed-methods feasibility cluster randomised controlled trial of the AMBER care bundle. Trials..

[49] McMillan SC, Small BJ, Haley WE (2011). Improving hospice outcomes through systematic assessment: a clinical trial. Cancer Nurs.

[50] Gilbert JE, Howell D, King S, Sawka C, Hughes E, Angus H (2012). Quality improvement in cancer symptom assessment and control: the provincial palliative care integration project (PPCIP). J Pain Symptom Manag.

[51] Mercadante S, Adile C, Aielli F, Lanzetta G, Mistakidou K, Maltoni M (2019). Personalized goal for dyspnea and clinical response in advanced cancer patients. J Pain Symptom Manag.

[52] Cox A, Illsley M, Knibb W, Lucas C, O'Driscoll M, Potter C (2011). The acceptability of e-technology to monitor and assess patient symptoms following palliative radiotherapy for lung cancer. Palliat Med.

[53] Hockley J, Watson J, Oxenham D, Murray SA (2010). The integrated implementation of two end-of-life care tools in nursing care homes in the UK: an in-depth evaluation. Palliat Med.

[54] Jennings LA, Zingmond D, Louie R, Tseng CH, Thomas J, O'Malley K (2016). Use of the physician orders for life-sustaining treatment among California nursing home residents. J Gen Intern Med.

[55] Krumm N, Larkin P, Connolly M, Rode P, Elsner F (2014). Improving dementia care in nursing homes: experiences with a palliative care symptom-assessment tool (MIDOS). Int J Palliat Nurs.

[56] Landi F, Onder G, Tua E, Carrara B, Zuccala G, Gambassi G (2001). Impact of a new assessment system, the MDS-HC, on function and hospitalization of homebound older people: a controlled clinical trial. J Am Geriatr Soc.

[57] Ratner E, Norlander L, McSteen K (2001). Death at home following a targeted advance-care planning process at home: the kitchen table discussion. J Am Geriatr Soc.

[58] Schamp R, Tenkku L (2006). Managed death in a PACE: pathways in present and advance directives. J Am Med Dir Assoc.

[59] Zafirau WJ, Snyder SS, Hazelett SE, Bansal A, McMahon S (2012). Improving transitions: efficacy of a transfer form to communicate patients' wishes. Am J Med Qual.

[60] McGlinchey T, Mason S, Coackley A, Roberts A, Maguire M, Sanders J (2019). Serious illness care Programme UK: assessing the ‘face validity’, applicability and relevance of the serious illness conversation guide for use within the UK health care setting. BMC Health Serv Res.

[61] Mills AC, Levinson M, Dunlop WA, Cheong E, Cowan T, Hanning J (2018). Testing a new form to document ‘goals-of-care’ discussions regarding plans for end-of-life care for patients in an Australian emergency department. Emerg Med Australas.

[62] Bouvette M, Fothergill-Bourbonnais F, Perreault A (2002). Implementation of the pain and symptom assessment record (PSAR).[Erratum appears in J Adv Nurs. 2003 Jun;42(6):647]. J Adv Nurs.

[63] Kmet LM, Lee RC, Cook L (2004). Standard quality assessment criteria for evaluating primary research papers from a variety of fields.

[64] Henson LA, Gao W, Higginson IJ, Smith M, Davies JM, Ellis-Smith C (2014). Emergency department attendance by patients with cancer in their last month of life: a systematic review and meta-analysis. J Clin Oncol.

[65] Lee L, Packer TL, Tang SH, Girdler S (2008). Self-management education programs for age-related macular degeneration: a systematic review. Australas J Ageing.

[66] Rohwer A, Booth A, Pfadenhauer L, Brereton L, Gerhardus A, Mozygemba K (2016). Guidance on the use of logic models in health technology assessments of complex interventions [online].

[67] Tulsky JA, Beach MC, Butow PN, Hickman SE, Mack JW, Morrison RS (2017). A research agenda for communication between health care professionals and patients living with serious illness. JAMA Intern Med.

[68] Bernacki R, Paladino J, Neville BA, Hutchings M, Kavanagh J, Geerse OP (2019). Effect of the serious illness care program in outpatient oncology: a cluster randomized clinical trial. JAMA Intern Med.

[69] Onishi E, Nakagawa S, Uemura T, Shiozawa Y, Yuasa M, Ito K, et al. Physicians' perceptions and suggestions for the adaptation of a US-based serious illness communication training in a non-US culture: a qualitative study. J Pain Symptom Manag. 2021;62(2):400-409.e3. 10.1016/j.jpainsymman.2020.11.035. 10.1016/j.jpainsymman.2020.11.035 PMC8244824 33290856

[70] Schnelle JF, Bates-Jensen BM, Chu L, Simmons SF (2004). Accuracy of nursing home medical record information about care-process delivery: implications for staff management and improvement. J Am Geriatr Soc.

[71] Chen J, Ou L, Hollis SJ (2013). A systematic review of the impact of routine collection of patient reported outcome measures on patients, providers and health organisations in an oncologic setting. BMC Health Serv Res.

[72] Chadborn NH, Goodman C, Zubair M, Sousa L, Gladman JRF, Dening T (2019). Role of comprehensive geriatric assessment in healthcare of older people in UK care homes: realist review. BMJ Open.

[73] Ellis G, Gardner M, Tsiachristas A, Langhorne P, Burke O, Harwood RH, et al. Comprehensive geriatric assessment for older adults admitted to hospital. Cochrane Database Syst Rev. 2017;9(9):CD006211. 10.1002/14651858.CD006211.pub3. 10.1002/14651858.CD006211.pub3 PMC6484374 28898390

[74] Rubenstein LZ, Siu AL, Wieland D (1989). Comprehensive geriatric assessment: toward understanding its efficacy. Aging Clin Exp Res.

[75] Hawes C, Morris JN, Phillips CD, Fries BE, Murphy K, Mor V (1997). Development of the nursing home resident assessment instrument in the USA. Age Ageing.

[76] Hirdes JP, Ljunggren G, Morris JN, Frijters DHM, Finne Soveri H, Gray L (2008). Reliability of the interRAI suite of assessment instruments: a 12-country study of an integrated health information system. BMC Health Serv Res.

[77] Hearn J, Higginson IJ (1999). Development and validation of a core outcome measure for palliative care: the palliative care outcome scale. Palliative Care Core Audit Project Advisory Group. Qual Health Care.

[78] Murtagh FE, Ramsenthaler C, Firth A, Groeneveld EI, Lovell N, Simon ST (2019). A brief, patient- and proxy-reported outcome measure in advanced illness: validity, reliability and responsiveness of the integrated palliative care outcome scale (IPOS). Palliat Med.

[79] Collins ES, Witt J, Bausewein C, Daveson BA, Higginson IJ, Murtagh FE (2015). A systematic review of the use of the palliative care outcome scale and the support team assessment schedule in palliative care. J Pain Symptom Manag.

