# Supplementary material for: Managing clinical uncertainty in older people towards the end of life: a systematic review of person-centred tools
Source: BMC Palliat Care. 2021 Oct 22;20:168. doi: 10.1186/s12904-021-00845-9 (PMC8532380; doi:10.1186/s12904-021-00845-9)
Supplement: Supplementary file 1 — Additional file 1. Conceptual framework. [file 12904_2021_845_MOESM1_ESM.docx]

# Additional file 1. Conceptual framework of uncertainty

Conceptual framework of uncertainty in older people towards the end of life adapted from Mishel, (9-11) Goodman et al, (13) and Etkind (12)

Definitions and examples types of uncertainty across levels of uncertainty

| Older person towards the end of life | |
| --- | --- |
| Level of engagement | The extent to which the person is involved in decisions about their own care, and impact of cognitive decline on level of engagement^6^ |
| Information preferences | The extent to which the person wishes to be involved in decisions about their care and treatment ranging from little/no involvement to fully involved |
| Temporal focus | Whether the person’s focus is on the present or future (or past)^6^ |
| Treatment uncertainty | |
| Complexity | Multi-morbidity frequently with cognitive impairment |
| Unpredictability | Resulting from frailty trajectory of cumulative deficit with unpredictability of recovery or further decline from minor health events |
| Ambiguity | Resulting from e.g. multi-morbidity and challenges in assessing and treating multiple symptom presentation with uncertain cause. Knowing how best to treat symptoms is often challenging. |
| Lack of information | Difficulties making decisions about treatment due to lack of information about e.g. functional baseline prior to event, PMH, patient preferences ‘what matters to you’. Challenges in assessing in people who have difficulties verbally communicating, particularly, if no advocate who knows the person well e.g. family member, care home staff. |
| Relational uncertainty | |
| Complexity | E.g. As a result of social concerns such as family or carer concerns or multiple family members with different priorities, social isolation, vulnerability; complexity due to multiple teams and multi-disciplinary and multi-professional teams; and how all work together |
| Unpredictability | Staff turnover, shift work, agency staff affect working relationships making care provision more challenging. The unpredictability of workforce affects relationships e.g. agency staff who do not know the person or other health professionals; carer stress or frailty or ill-health. |
| Ambiguity | Multiple agencies and disciplines involved in care resulting in lack of clarity about roles and responsibilities, tensions between priorities e.g. family and person |
| Lack of information | Communication challenges including challenges of cognitively impaired person to communicate their wishes, challenges of communication between all agencies involved in care; challenges of communication with multiple family members |
| Service uncertainty | |
| Complexity | Multiple services including health and social care; different budgets and funding requirements |
| Unpredictability | Staff changes including shift work, staff turnover, staff shortages; availability of services e.g. home care resulting in discharge delays. Unpredictability of workforce affects resources and care provision |
| Ambiguity | Conflicts over care provision, place of care, tensions between different services and budgets |
| Lack of information | Lack of shared documentation between services; poor access to information such as multiple IT systems. |
