# Supplementary material for: Managing clinical uncertainty in older people towards the end of life: a systematic review of person-centred tools
Source: BMC Palliat Care. 2021 Oct 22;20:168. doi: 10.1186/s12904-021-00845-9 (PMC8532380; doi:10.1186/s12904-021-00845-9)
Supplement: Supplementary file 3 — Additional file 3. Full search strategy. [file 12904_2021_845_MOESM3_ESM.docx]

# Additional file 3. Full search strategy

| Medline  (Ovid Medline(r) 2000 - December 2019) | PsycINFO  (Ovid-Kings 2000 -December 2019) | CINAHL  (CINAHL – Kings)  2000 – December 2019 | IBSS  (ProQuest)  2000 – July 2018 |
| --- | --- | --- | --- |
| Population MeSH  1. exp chronic disease/  2. exp terminally ill/  3. exp terminal care/  4. palliative care/  5. frailty/  6. exp comorbidity/  7. exp frail elderly/  Population Key  8. chronic disease.tw.  9. EoL.tw.  10. End?of?life.tw.  11. dying.tw.  12. palliative.tw.  13. (last adj4 life).tw.  14. hospice.tw.  15. life limit*.tw.  16. advanced disease*.tw.  17. palliative treatment.tw.  18. palliative medicine.tw.  19. terminal care.tw.  20. terminally ill.tw.  21. end-of-life care.tw.  22. hospice care.tw.  23. palliation.tw.  24. palliative care*.tw.  25. 1 or 2 or 3 or 4 or 5 or 6 or 7 or 8 or 9 or 10 or 11 or 12 or 13 or 14 or 15 or 16 or 17 or 18 or 19 or 20 or 21 or 22 or 23 or 24  Uncertainty MeSH  26. uncertainty/  27. information seeking behavior/  28. Communication Barriers/  29. physician-nurse relations/  30. Professional-Family Relations/  31. interdisciplinary communication/  32. nurse-patient relations/  33. physician-patient relations/  34. exp disease progression/  35. exp recurrence/  36. watchful waiting/  37. patient discharge/  38. exp aftercare/  39. patient handoff/  40. patient transfer/  41. patient readmission/  42. progressive patient care/  43. Exp decision making  44. critical care/  45. long-term care/  46. exp nursing homes/  47. homes for the aged/  48. exp emergency service, hospital/  Uncertainty Key  49. ambigu*.tw.  50. unpredictab*.tw.  51. inconsisten*.tw.  52. deteriorat*.tw.  53. uncertain*.tw.  54. fluctuat*.tw.  55. transit*.tw.  56. long term care.tw.  57. residential care home*.tw.  58. (decision adj2 making).tw  59. 26 or 27 or 28 or 29 or 30 or 31 or 32 or 33 or 34 or 35 or 36 or 37 or 38 or 39 or 40 or 41 or 42 or 43 or 44 or 45 or 46 or 47 or 48 or 49 or 50 or 51 or 52 or 53 or 54 or 55 or 56 or 57 or 58    Tools MeSH  60. nursing assessment/  61. symptom assessment/    62. geriatric assessment/  63. needs assessment/  64. exp patient care planning/  65. Health Communication/  66. hospital communication systems/  67. patient care bundles/  68. decision support systems, clinical/  69. decision support techniques/  70. patient health questionnaire/  71. self report/  72. Exp patient-centred care    Tools Key  73. assess* instrument.tw.  74. assess* tool.tw.  75. health communication.tw.  76. (co?ordination adj3 care).tw.  77. communicat*.tw.  78. (discharg* adj3 plan*).tw.  79. (psycholog* adj3 plan*).tw.  80. (psychosocial adj3 assess).tw.  81. (Comprehensive adj3 assessment).tw  82.Outcome measure.tw  83. 60 or 61 or 62 or 63 or 64 or 65 or 66 or 67 or 68 or 69 or 70 or 71 or 72 or 73 or 74 or 75 or 76 or 77 or 78 or 79 or 80 or 81 or 82  Outcome MeSH  84. exp quality of life/  85. personal satisfaction/  86. exp activities of daily living/  87. exp mental health/  98. exp social isolation/  89. exp social support/  90. exp patient satisfaction/  91. personhood/    Outcome Key  92. good death.tw.  93. symptom*.tw.  94. concern*.tw.  95. attainment.tw.  96. dignity.tw.  97. quality of life.tw.  98. qol.tw.  99. (quality adj2 life).tw.  100. distress.tw.  101. wellbeing.tw.  102. adl*.tw.  103. activities of daily living.tw.  104. symptom management.tw.  105. psychosocial.tw.  106. (psycho adj social).tw.  107. psychological distress.tw.  108. enablement.tw.  109. mastery.tw.  120. resilience.tw.  121. stable.tw.  122. personhood.tw.  123. 84 or 85 or 86 or 87 or 88 or 89 or 90 or 91 or 92 or 93 or 94 or 95 or 96 or 97 or 98 or 99 or 100 or 101 or 102 or 103 or 104 or 105 or 106 or 107 or 108 or 109 or 110 or 111 or 112 or 113 or 114 or 115 or 116 or 117 or 118 or 119 or 120 or 121 or 122  124. 25 and 59 and 83 and 123  125. limit 124 to yr="2000 -Current" | Population MeSH  1. exp chronic illness/  2. exp terminally ill/  3. exp terminal care/  4. palliative care/  5. Health Impairments/  6. exp comorbidity/  7. exp Geriatric Patients/  Population Key  8. chronic disease.tw.  9. EoL.tw.  10. End of life.tw.  11. dying.tw.  12. palliative.tw.  13. (last adj4 life).tw.  14. hospice.tw.  15. life limit*.tw.  16. advanced disease*.tw.  17. palliative treatment.tw.  18. palliative medicine.tw.  19. terminal care.tw.  20. terminally ill.tw.  21. end-of-life care.tw.  22. hospice care.tw.  23. palliation.tw.  24. palliative care*.tw.  25. 1 or 2 or 3 or 4 or 5 or 6 or 7 or 8 or 9 or 10 or 11 or 12 or 13 or 14 or 15 or 16 or 17 or 18 or 19 or 20 or 21 or 22 or 23 or 24  Uncertainty MeSH  26. uncertainty/  27. information seeking/  28. Communication Barriers/  29. physician-nurse relations/  30. Professional-Family Relations/  31. interdisciplinary communication/  32. nurse-patient relations/  33. physician-patient relations/  34. exp disease progression/  35. exp recurrence/  36. watchful waiting/  37. patient discharge/  38. exp aftercare/  39. patient handoff/  40. patient transfer/  41. patient readmission/  42. progressive patient care/  43. Exp decision making  44. critical care/  45. long-term care/  46. exp nursing homes/  47. homes for the aged/  48. exp emergency service, hospital/  Uncertainty Key  49. ambigu*.tw.  50. unpredictab*.tw.  51. inconsisten*.tw.  52. deteriorat*.tw.  53. uncertain*.tw.  54. fluctuat*.tw.  55. transit*.tw.  56. long term care.tw.  57. residential care home*.tw.  58. (decision adj2 making).tw  59. 26 or 27 or 28 or 29 or 30 or 31 or 32 or 33 or 34 or 35 or 36 or 37 or 38 or 39 or 40 or 41 or 42 or 43 or 44 or 45 or 46 or 47 or 48 or 49 or 50 or 51 or 52 or 53 or 54 or 55 or 56 or 57 or 58    Tools MeSH  60. nursing assessment/  61. symptom assessment/    62. geriatric assessment/  63. needs assessment/  64. exp patient care planning/  65. Health Communication/  66. hospital communication systems/  67. patient care bundles/  68. Decision Support Systems/  69. decision support techniques/  70. patient health questionnaire/  71. self report/  72. Exp patient-centred care    Tools Key  73. assess* instrument.tw.  74. assess* tool.tw.  75. health communication.tw.  76. (co?ordination adj3 care).tw.  77. communicat*.tw.  78. (discharg* adj3 plan*).tw.  79. (psycholog* adj3 plan*).tw.  80. (psychosocial adj3 assess).tw.  81. (Comprehensive adj3 assessment).tw  82.Outcome measure.tw  83. 60 or 61 or 62 or 63 or 64 or 65 or 66 or 67 or 68 or 69 or 70 or 71 or 72 or 73 or 74 or 75 or 76 or 77 or 78 or 79 or 80 or 81 or 82  Outcome MeSH  84. exp quality of life/  85. personal satisfaction/  86. exp activities of daily living/  87. exp mental health/  98. exp social isolation/  89. exp social support/  90. exp patient satisfaction/  91. personhood/    Outcome Key  92. good death.tw.  93. symptom*.tw.  94. concern*.tw.  95. attainment.tw.  96. dignity.tw.  97. quality of life.tw.  98. qol.tw.  99. (quality adj2 life).tw.  100. distress.tw.  101. wellbeing.tw.  102. adl*.tw.  103. activities of daily living.tw.  104. symptom management.tw.  105. psychosocial.tw.  106. (psycho adj social).tw.  107. psychological distress.tw.  108. enablement.tw.  109. mastery.tw.  120. resilience.tw.  121. stable.tw.  122. personhood.tw.  123. 84 or 85 or 86 or 87 or 88 or 89 or 90 or 91 or 92 or 93 or 94 or 95 or 96 or 97 or 98 or 99 or 100 or 101 or 102 or 103 or 104 or 105 or 106 or 107 or 108 or 109 or 110 or 111 or 112 or 113 or 114 or 115 or 116 or 117 or 118 or 119 or 120 or 121 or 122  124. 25 and 59 and 83 and 123  125. limit 124 to yr="2000 -Current" | Population MeSH  1. MW “chronic disease”  2. MW “terminally ill”  3. MW “terminal care”  4. MW “palliative care”  5. MW “frailty”  6. MW “comorbidity”  7. MW “frail elderly”  Population Key (TX)  8. TX “chronic disease”  9. TX “EoL”  10. TX “End of life”  11. TX “dying”  12. TX “palliative”  13. TX “last N4 life”  14. TX “hospice”  15. TX “life limit”  16. TX “advanced disease”  17. TX “palliative treatment”  18. TX “palliative medicine”  19. TX “terminal care”  20. TX “terminally ill”  21. TX “end-of-life care”  22. TX “hospice care”  23. TX “palliation”  24. TX “palliative care”  25. 1 or 2 or 3 or 4 or 5 or 6 or 7 or 8 or 9 or 10 or 11 or 12 or 13 or 14 or 15 or 16 or 17 or 18 or 19 or 20 or 21 or 22 or 23 or 24  Uncertainty MeSH  26. MW “uncertainty”  27. MW “information seeking behavior”  28. MW “Communication Barriers”  29. MW “physician-nurse relations”  30. MW “Professional-Family Relations”  31. MW “interdisciplinary communication”  32. MW “nurse-patient relations”  33. MW “physician-patient relations”  34. MW “disease progression”  35. MW “recurrence”  36. MW “watchful waiting”  37. MW “patient discharge”  38. MW “aftercare”  39. MW “patient handoff”  40. MW “patient transfer”  41. MW “patient readmission”  42. MW “progressive patient care”  43. MW (“Exp decision making”+)  44. MW “critical care”  45. MW “long-term care”  46. MW “nursing homes”  47. “homes for the aged”  48. “emergency service, hospital”  Uncertainty Key  49. TX “ambigu*”  50. TX “unpredictab*”  51. TX “inconsisten*” 52. TX “deteriorate*”  53. TX “uncertain*”  54. TX “fluctuat*”  55. TX “transit*”  56. TX “long term care”  57. TX “residential care home”  58. TX “decision N2 making”  59. 26 or 27 or 28 or 29 or 30 or 31 or 32 or 33 or 34 or 35 or 36 or 37 or 38 or 39 or 40 or 41 or 42 or 43 or 44 or 45 or 46 or 47 or 48 or 49 or 50 or 51 or 52 or 53 or 54 or 55 or 56 or 57 or 59  Tools MeSH  60. MW “nursing assessment”  61. MW “symptom assessment”  62. MW “geriatric assessment”  63. MW “needs assessment”  64. MW “patient care planning”  65. MW “Health Communication”  66. MW “hospital communication systems”  67. MW “patient care bundles”  68. MW “decision support systems, clinical”  69. MW “decision support techniques”  70. MW “patient health questionnaire”  71. MW “self report”  72. MW “patient-centred care”  Tools Key  73. TX “assess* instrument”  74. TX “assess* tool”  75. TX “health communication”  76. TX “co-ordination N3 care”  77. TX “communicat*”  78. TX “discharg* N3 plan*”  79. TX“psycholog* N3 assess*”  80. TX “psychosocial N3 assess”  81. TX “Comprehensive N3 assessment”  82. TX “Outcome measure”  83. 60 or 61 or 62 or 63 or 64 or 65 or 66 or 67 or 68 or 69 or 70 or 71 or 72 or 73 or 74 or 75 or 76 or 77 or 78 or 79 or 80 or 81 or 82  Outcome MeSH  84. MW “quality of life”  85. MW “personal satisfaction”  86. MW “activities of daily living”  87. MW “mental health”  88. MW “social isolation”  89. MW “social support”  90. MW “patient satisfaction”  91. MW “personhood”  Outcome Key  92. TX “good death”  93. TX “symptom”  94. TX “concern”  95. TX “attainment”  96. TX “dignity”  97. TX “quality of life”  98. TX “qol”  99. “quality N2 life”  100. TX “distress”  101. TX “wellbeing”  102. TX “adl”  103. TX “activities of daily living”  104. TX “symptom management”  105. TX “psychosocial”  106. TX “psycho N0 social”  107. TX “psychological distress”  108. TX “enablement”  109. TX “mastery”  110. TX “resilience”  111. TX “stable”  112. TX “personhood”  113. 84 or 85 or 86 or 87 or 88 or 89 or 90 or 91 or 92 or 93 or 94 or 95 or 96 or 97 or 98 or 99 or 100 or 101 or 102 or 103 or 104 or 105 or 106 or 107 or 108 or 109 or 110 or 111 or 112  114. 1 and 2 and 3 and 4  115. Limit 5 to yr=”2000 –Current” | Population MeSH  1.((chronic disease) or  (terminally ill) or  (terminal care) or  (palliative care) or  (frailty) or  (comorbidity) or  (frail elderly) or  Population Key  (chronic disease) or  (EoL) or  (End of life) or  (dying) or  (palliative) or  (last adj4 life) or  (hospice) or  (life limit) or  (advanced disease) or  (palliative treatment) or  (palliative medicine) or  (terminal care) or  (terminally ill) or  (end-of-life care) or  (hospice care) or  (palliation) or  (palliative care))  Uncertainty MeSH  2. ((uncertainty) or  (information seeking behavior) or  (Communication Barriers) or  (physician-nurse relations) or  (Professional-Family Relations) or  (interdisciplinary communication) or  (nurse-patient relations) or  (physician-patient relations) or  (disease progression) or  (recurrence) or  (watchful waiting) or  (patient discharge) or  (aftercare) or  (patient handoff) or  (patient transfer) or  (patient readmission) or  (progressive patient care) or  (Exp decision making) or  (critical care) or  (long-term care) or  (nursing homes) or  (homes for the aged) or  (emergency service, hospital) or  Uncertainty Key  (ambigu) or  (unpredictab) or  (inconsisten) or  (deteriorat) or  (uncertain) or  (fluctuat) or  (transit) or  (long term care) or  (residential care home) or  (decision adj2 making))  Tools MeSH  3. ((nursing assessment) or  (symptom assessment) or  (geriatric assessment) or  (needs assessment) or  (patient care planning) or  (Health Communication) or  (hospital communication systems) or  (patient care bundles) or  (decision support systems, clinical) or  (decision support techniques) or  (patient health questionnaire) or  (self report) or  (patient-centred care) or  Tools Key  (assess instrument) or  (assess tool) or  (health communication) or  (co-ordination adj3 care) or  (communicat) or  (discharg adj3 plan) or  (psycholog adj3 assess) or  (psychosocial adj3 assess)) or  (Comprehensive adj3 assessment) or  (Outcome measure))  Outcome MeSH  4. ((quality of life) or  (personal satisfaction) or  (activities of daily living) or  (mental health) or  (social isolation) or  (social support) or  (patient satisfaction) or  (personhood) or  Outcome Key  (good death) or  (symptom) or  (concern) or  (attainment) or  (dignity) or  (quality of life) or  (qol) or  (quality adj2 life) or  (distress) or  (wellbeing) or  (adl) or  (activities of daily living) or  (symptom management) or  (psychosocial) or  (psycho adj social) or  (psychological distress) or  (enablement) or  (mastery) or  (resilience) or  (stable)  (personhood))  5. 1 and 2 and 3 and 4  6. Limit 5 to yr=2000 –Current |
